# Membrane vesicle delivery of a streptococcal M protein disrupts the blood–brain barrier by inducing autophagic endothelial cell death

**DOI:** 10.1073/pnas.2219435120

**Published:** 2023-06-05

**Authors:** Fei Pan, Mingli Zhu, Ying Liang, Chen Yuan, Yu Zhang, Yuchang Wang, Hongjie Fan, Matthew K. Waldor, Zhe Ma

**Affiliations:** ^a^Ministry of Agriculture Key Laboratory of Animal Bacteriology, the International Joint Laboratory of Animal Health and Food Safety, and College of Veterinary Medicine, Nanjing Agricultural University, Nanjing, Jiangsu 210095, China; ^b^Jiangsu Co-innovation Center for Prevention and Control of Important Animal Infectious Diseases and Zoonoses, Yangzhou 225009, China; ^c^HHMI, Boston, MA 02115; ^d^Brigham and Women’s Hospital Division of Infectious Diseases, Boston, MA 02115; ^e^Department of Microbiology, Harvard Medical School, Boston, MA 02115

**Keywords:** Streptococcal M proteins, membrane vesicles, *Streptococcus equi subsp. zooepidemicus*, blood–brain barrier, PTEN, autophagic cell death

## Abstract

M family proteins are critical virulence determinants of Streptococci. *Streptococcus equi* subsp. *zooepidemicus* (SEZ) are Group C streptococci that cause meningitis in animals and humans. SzM, the M protein of SEZ, has been linked to SEZ brain invasion. Here, we demonstrate that SzM is important in SEZ disruption of the blood–brain barrier (BBB). SEZ release SzM-bound membrane vesicles (MVs), and endocytosis of these vesicles by human brain endothelial microvascular cells (hBMECs) results in SzM-dependent cytotoxicity. Furthermore, administration of SzM-bound MVs disrupted the murine BBB. A CRISPR screen revealed that SzM cytotoxicity in hBMECs depends on PTEN-related activation of autophagic cell death. Pharmacologic inhibition of PTEN activity prevented SEZ disruption of the murine BBB and delayed mortality. Our data show that MV delivery of SzM to host cells plays a key role in SEZ pathogenicity and suggests that MV delivery of streptococcal M family proteins is likely a common streptococcal virulence mechanism.

## Significance

*Streptococcus equi* subsp. *zooepidemicus* (SEZ) are Group C streptococci that cause meningitis in animals and humans. Here, we show SEZ releases membrane vesicles (MVs) that contain the SEZ M protein, SzM. MV provides an efficient means of delivery of SzM to host cells. Endocytosis of these vesicles results in autophagic cell death in hBMECs (human brain endothelial microvascular cells) and disruption of the BBB (blood–brain barrier) in mice. Blockade of MV endocytosis or inactivation of autophagic death attenuated SEZ pathogenicity in mice. Together, these findings provide therapeutic targets for treatment of SEZ infection and extend our knowledge of streptococcal virulence mechanisms.

*Streptococcus equi* subsp. *zooepidemicus* (SEZ) belongs to Lancefield group C streptococci (GCS) and is well known as a zoonotic pathogen frequently isolated from horses (1). Human- and swine-isolated strains in the multilocus sequence typing cluster ST194 have been identified as hypervirulent (2–4). For example, a 2011 outbreak of SEZ ST194 in humans associated with raw pork consumption in Thailand was fatal in 6 out of 14 patients (5). An outbreak of SEZ ST194 in 2019 in North American pig farms led to the sudden death of thousands of pigs (3, 6). Meningitis is a very common clinical manifestation caused by SEZ infection in both humans and pigs (7–11). In experimental infection of pigs with swine-isolated SEZ ST194, 80% of animals had high SEZ burdens in their cerebrospinal fluid (CSF), suggesting that this pathogen is capable of disrupting the blood–brain barrier (BBB) and infecting the central nervous system (CNS) (4).

The BBB is an effective biological barrier that separates the brain from the rest of the body. The barrier is mainly formed by microvascular endothelial cells and astrocytes and provides a significant obstacle to the entry of not only drugs but also pathogens into the brain (12, 13). Nevertheless, streptococci have developed varied strategies to disrupt this barrier and invade the brain (14–17). The BifA protein in SEZ ST194 contributes to BBB disruption, but even in its absence, SEZ can still enter the CNS (18). In a murine infection model, a *szm*-deficient SEZ strain had an ~1,000-fold lower burden in the brain 24 h after intravenous (i.v.) injection, raising the possibility that the *szm*-encoded M protein (SEZ M protein, SzM) also contributes to SEZ brain invasion (19).

M proteins were initially described as surface-associated, highly versatile molecules in group A streptococcus (GAS) (20). M family proteins are usually anchored to the cell wall on the bacterial surface and most studies of their functions have focused on recruitment of host molecules, such as IgG, C4BP, fibrinogen, and factor H, to the bacterial surface or on bacterial adhesion to host cells (21). GCS is closely related to GAS and encodes a surface-associated M protein SzM that shares a similar alpha-helical fibrillar structure with M proteins, but with low sequence identity (22, 23). SzM proteins, like M proteins, also contain a variable region that exhibits sequence heterogeneity in different strains and can be used to classify SEZ strains into M types (24). Interestingly, the SzM protein in swine-isolated SEZ ST194 strains, regardless of the geographic sites of their isolation [e.g., North America (TN-74079 and OH71905) or China (ATCC35246, CY)], have 100% identity, suggesting that this SzM protein variant is associated with the hypervirulent swine-isolated SEZ strains (3).

Although the SzM protein is present in supernatants of hypervirulent SEZ cultures, the mechanism of its release from the bacterial surface and the contribution of nonsurface-associated SzM protein to SEZ pathogenicity are both unknown. Numerous gram-positive pathogens, including streptococci, release extracellular vesicles, termed membrane vesicles (MVs, also called CMVs for cytoplasmic MVs) (25, 26). Various membrane-associated virulence factors have been associated with MVs; e.g., *Staphylococcus aureus* MVs contain protein A (relevant to immune evasion) (27). MVs can deliver bacterial cargos to host cells, facilitating the entrance of proteins such as cytotoxins that lack export signals into host cells (28). In streptococci, there is little direct evidence suggesting that M family proteins associate with MVs (29). Moreover, the relevance of MV-borne M proteins to streptococcal pathogenicity is absent.

Here, we show that SzM is shed in SEZ MVs. MV-borne SzM is cytotoxic to human brain microvascular endothelial cells (hBMECs) and contributes to SEZ pathogenicity in a murine model by promoting BBB disruption. Blocking endocytosis reduced cytotoxicity and BBB disruption caused by SzM-bound MVs. A genome-scale CRISPR/Cas9 screen suggested that SzM induces PTEN (phosphatase and tensin homolog)-related autophagy-dependent cell death in hBMECs. Moreover, inhibition of PTEN activation protected the murine BBB from SzM-bound MVs toxicity and disruption. Collectively, our findings suggest that SEZ release of MV-borne SzM plays a critical role in SEZ pathogenicity and point to therapeutic approaches for SEZ infections.

## Results

### *szm* Contributes to SEZ BBB Disruption in Mice.

**Fig. 1.**
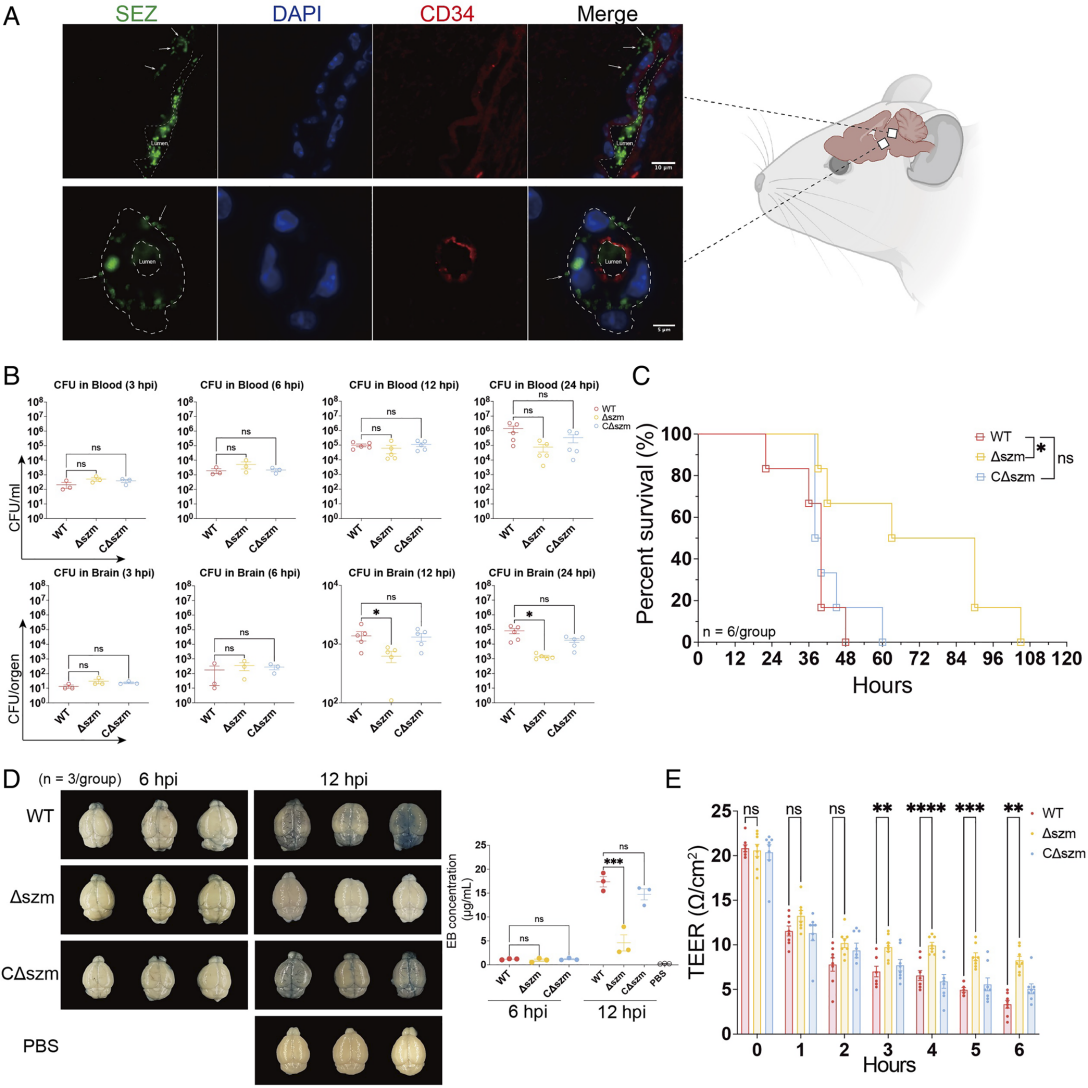
s*zm* contributes to SEZ virulence and BBB penetration by increasing BBB permeability. (*A*) Immunofluorescent staining of sections from mouse brains from animals challenged with 1 × 10^6^ CFU of SEZ via the tail vein; bacteria were labeled with anti-SzM monoclonal antibody (green), endothelial cells were labeled with CD34 monoclonal antibody (red), and cell nuclei were stained with DAPI (blue). (*B*–*D*) Mice were challenged with 5 × 10^6^ CFU WT SEZ, CΔszm, or 1 × 10^7^ CFU Δszm via i.v. injection through tail vein; (*B*) The bacterial burden in the blood and brain at 3, 6, 12, and 24 hpi (* indicates *P* < 0.05 and ns indicates no significant difference with one-way ANOVA); (*C*) Survival curves of animals challenged with the indicated strains. (* indicates *P* < 0.05 and ns indicates no significant difference with the log-rank test vs. WT); (*D*) Evans Blue (EB) dye accumulation at 6 and 12 hpi to evaluate the permeability of the BBB. The brains were dissected and EB was extracted and quantified (*** indicates *P* < 0.001 and ns indicates no significant difference with one-way ANOVA). (*E*) Coincubation of indicated SEZ strains with a tissue culture endothelium monolayer model (MOI = 1:10); TEER values were measured at the indicated time points (n = 8, ** indicates *P* < 0.01, *** indicates *P* < 0.001, **** indicates *P* < 0.0001, and ns indicates no significant difference with two-way ANOVA).

Intravenous inoculation of SEZ through the mouse tail vein routinely gives rise to bacteremia and meningitis (18), suggesting that this pathogen encodes mechanisms that facilitate its invasion of the CNS. Eighteen hours post-i.v. inoculation, SEZ could be identified on both sides of the blood vessel endothelial (or pericyte) layer in the brain consistent with the idea SEZ can penetrate CNS blood vessels, traversing the BBB (Fig. 1*A*). To investigate the contribution of *szm* to SEZ CNS invasion, a *szm-*deficient mutant strain (Δszm) and a complemented strain (CΔszm) were constructed and verified (*SI Appendix*, Fig. S1 *A* and *B*). The wild-type (WT) and Δszm mutant exhibited similar growth in THB (Todd Hewitt Broth) medium and in porcine CSF (*SI Appendix*, Fig. S1 *C* and *D*), suggesting that the mutant is not impaired for growth in CSF. However, the Δszm mutant had defective survival in blood (30). Thus, to compare the capacity of WT and Δszm SEZ strains to cross the BBB and proliferate in the brain, animals were inoculated with ~2× greater colony-forming unit (CFU) of the mutant vs. the WT bacteria. At this dose, there were similar recoverable CFU of both strains from blood for at least the first 24 h of infection (Fig. 1*B*). During the first 6 h following inoculation, the numbers of WT and Δszm CFU recovered from the brain were also similar. However, by 12 hours postinfection (hpi), the brain CFU burden of the WT strain exceeded that of the Δszm mutant, and the differences became more marked at 24 hpi, suggesting that the defect of the Δszm mutant to penetrate the BBB and/or proliferate in the brain becomes more pronounced as the duration of infection increases. Furthermore, despite the larger dose, the Δszm challenged mice survived 50 h longer than mice challenged with the WT strain. Complementation of the *szm* gene in the Δszm mutant restored its CFU burden in the CNS and partially restored its lethality (Fig. 1 *B* and *C*), confirming the importance of *szm* in SEZ virulence.

An Evans Blue (EB) dye-based assay (31) was used to assess the permeability of the BBB during SEZ infection. At 6 hpi, little EB was observed in the brains of animals inoculated with either the WT or the Δszm mutant, suggesting that the BBB remains intact at this point (Fig. 1*D*). However, at 12 hpi, in accord with differences in brain CFU burden data, greater amounts of EB were observed in the brains of WT vs. Δszm infected animals, suggesting that there is a correlation between BBB permeability and CFU burden in the brain (Fig. 1 *B* and *D*). Thus, the lower CFU burden of the Δszm mutant vs. the WT strain in the brain by 12 hpi may at least in part be due to an impaired capacity of the mutant to disrupt the BBB.

We also used a tissue culture-based in vitro endothelium monolayer model to partially simulate the BBB, where transendothelial electrical resistance (TEER) was used as an indicator of the integrity of the cellular barrier (32), to further explore the role of SzM in impairing the integrity of the BBB. For the first 2 h of infection in this model, both the WT SEZ and Δszm mutant reduced TEER, but by 3 h, the WT reduced TEER more than the Δszm mutant (*P* = 0.0013, Fig. 1*E*). Together, these observations suggest that *szm* contributes to SEZ’s capacity to increase the permeability of the BBB, facilitating bacterial penetration of this barrier after an initial period when infection becomes established.

### SzM Cytotoxicity Is Largely Dependent on Its Entry into Host Cells.

**Fig. 2.**
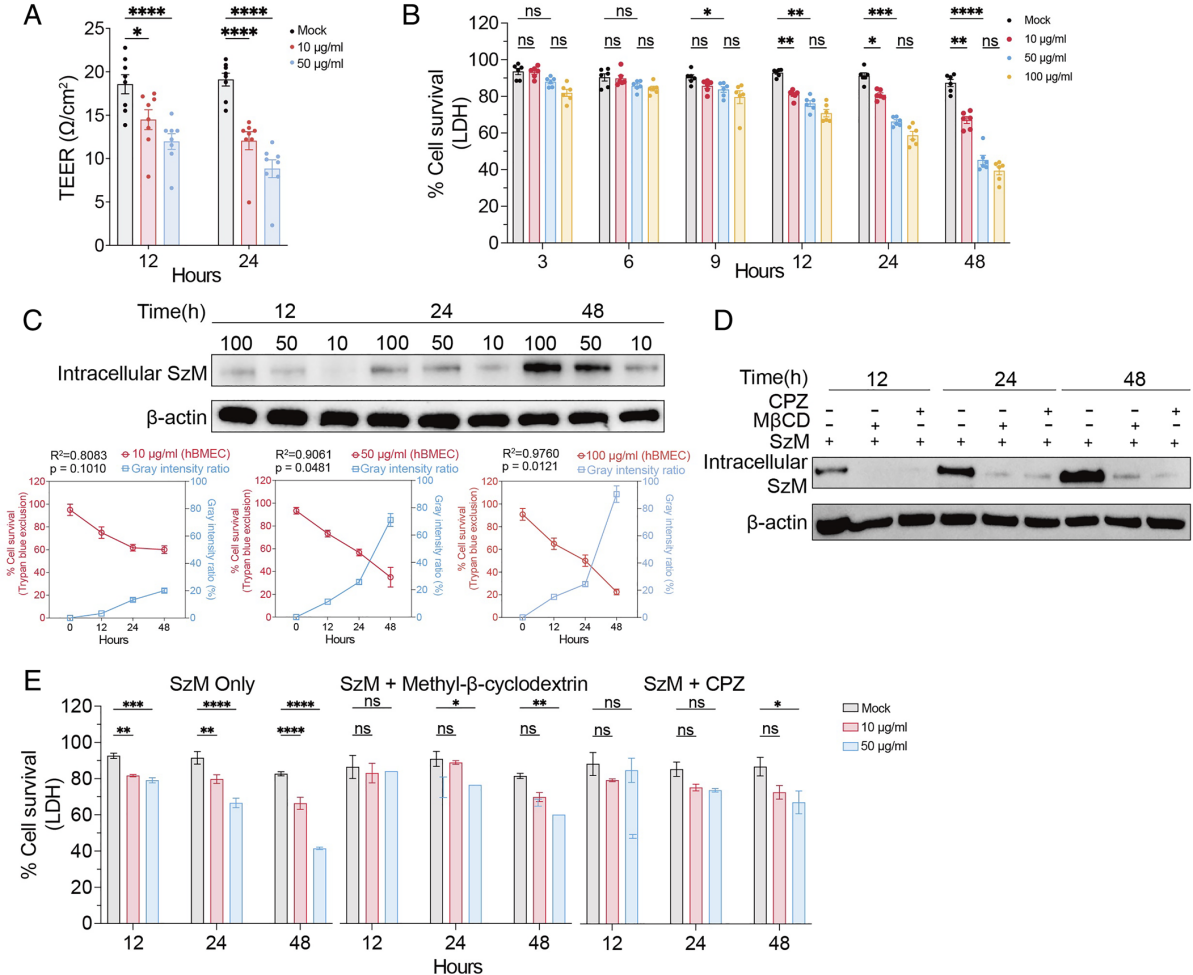
Intracellular SzM is cytotoxic for hBMECs. (*A*) Recombinant SzM protein or PBS (Mock) were applied to a tissue culture endothelium monolayer model and TEER values were measured 12 and 24 h later (n = 8, * indicates *P* < 0.05 and **** indicates *P* < 0.0001 with two-way ANOVA). (*B*) Time course of the survival of hBMECs after treatment with the indicated SzM protein concentrations; PBS was used in the mock group (n = 6, * indicates *P* < 0.05; ** indicates *P* < 0.01; **** indicates *P* < 0.0001, and ns indicates no significant difference with two-way ANOVA). (*C*) Intracellular SzM amounts in hBMECs assayed by immunoblot. Cells were harvested at 12, 24, and 48 h after the addition of the SzM protein (10, 50, 100 μg/mL), β-actin was used as a reference protein. The gray intensity was measured with ImageJ Fiji. The correlation between the gray intensity of the SzM/β-actin ratio and the percentage of cell survival (measured by trypan blue) was calculated with linear regression. (*D*) Intracellular accumulation of SzM in hBMECs after treatment with endocytosis inhibitor methyl-β-cyclodextrin (MβCD) or chlorpromazine (CPZ). The amount of intracellular SzM was detected by immunoblotting 12, 24, and 48 h after treatment with SzM protein (50 μg/mL). (*E*) Survival of hBMECs assessed with LDH release after treatment with SzM protein (10 or 50 μg/mL) and endocytosis inhibitors. PBS was the mock treatment. (n = 3, * indicates *P* < 0.05, ** indicates *P* < 0.01, *** indicates *P* < 0.001, **** indicates *P* < 0.0001, and ns indicates no significant difference with two-way ANOVA).

In the tissue culture model, recombinant SzM protein was also able to disrupt the TEER of the tissue culture endothelium monolayer model by increasing its permeability (Fig. 2*A*). SzM caused cytotoxicity to hBMECs in a time- and dosage-dependent manner (Fig. 2*B*). While 50 μg/mL of SzM protein resulted in greater cytotoxicity than 10 μg/mL, the 100 μg/mL dose did not lead to significantly increased cytotoxicity compared with 50 μg/mL (Fig. 2*B*), suggesting that the dose-dependent cytotoxicity of SzM is saturable. In principle, SzM disruption of TEER and cytotoxicity could be attributable to its action either outside or inside of host cells. In support of the latter possibility, SzM cytotoxicity generally increased along with its accumulation inside hBMECs (Fig. 2*C*).

Transfection experiments also supported the idea that SzM cytotoxic activity is manifest when it is present inside host cells. Following introduction of a plasmid expressing SzM fused with green fluorescent protein (SzM-GFP) in hBMECs (*SI Appendix*, Fig. S2*A*), a dim fluorescent signal became apparent 18 h later, along with SzM-GFP protein expression and by 22 h after transfection, cell death was apparent (*SI Appendix*, Fig. S2*B* and Movie S1). In contrast, hBMECs transfected with a plasmid expressing GFP alone exhibited bright GFP fluorescence and remained viable for the 24 h observation period (*SI Appendix*, Fig. S2*B* and Movie S2). The intracellular expression of SzM-GFP lead to ~50% cell death at 48 h, whereas ~80% of cells expressing GFP alone were alive at that point (*SI Appendix*, Fig. S2*C*). These data indicate that intracellular SzM can exert cytotoxicity.

Pharmacologic blockade of the cellular entry of SzM into hBMECs with inhibitors of clathrin-mediated endocytosis (chlorpromazine, CPZ) or lipid raft-mediated endocytosis (methyl-β-cyclodextrin), were both effective at preventing the intracellular accumulation of SzM; even after a 48-h incubation, little intracellular SzM was detected in hBMECs when endocytosis was inhibited (Fig. 2*D*). Both of these treatments also inhibited SzM cytotoxicity (Fig. 2*E*), bolstering the idea that SzM cytotoxicity in hBEMCs can primarily be attributed to its entry and accumulation within cells.

### SEZ MVs Harbor SzM.

**Fig. 3.**
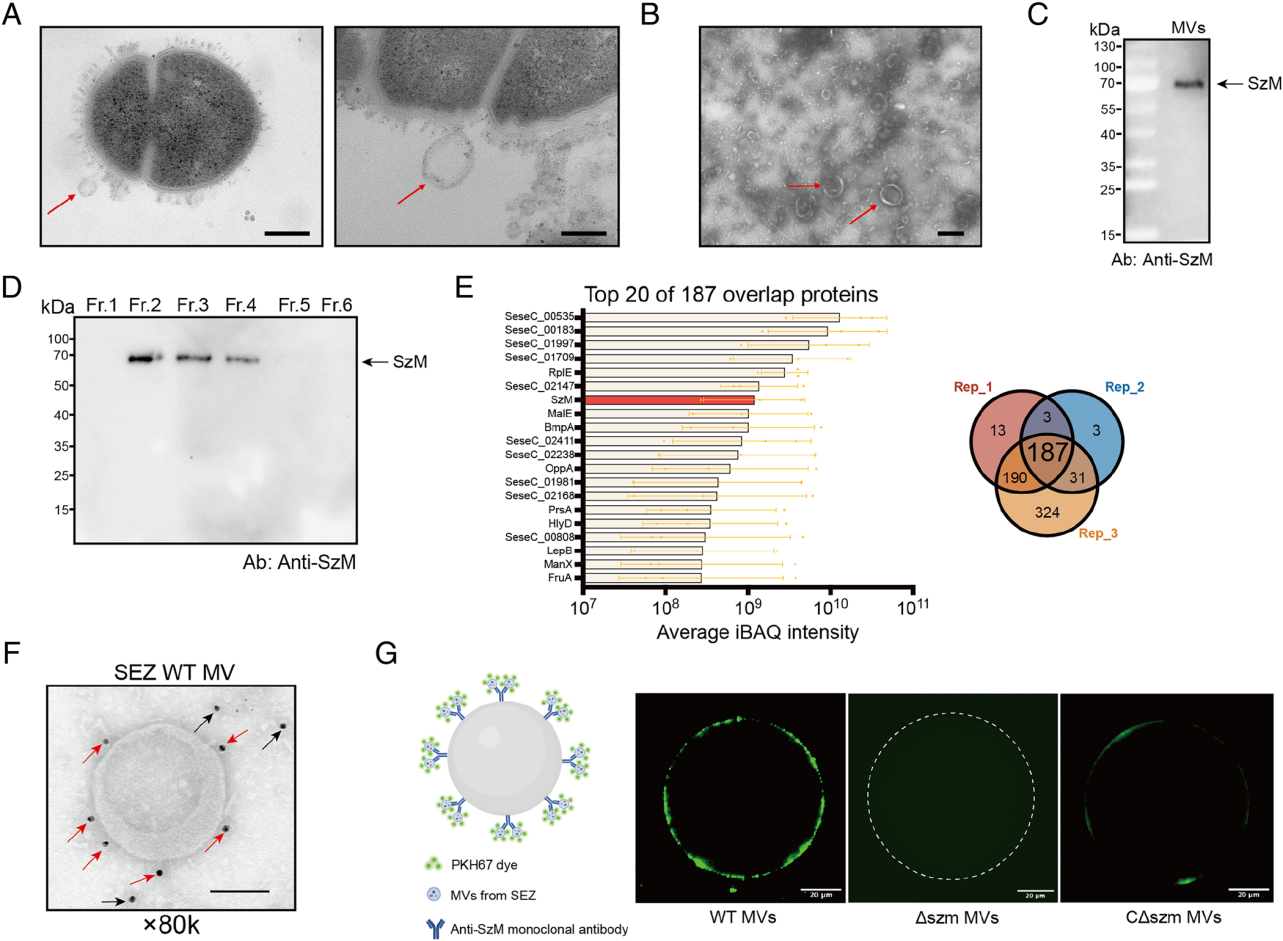
SzM is associated with SEZ membrane vesicles. (*A*) Transmission electron microscopy (TEM) of ultrathin sections of SEZ. The MVs (indicated by red arrows) appear to be attached to SEZ cells (scale bar, 200 nm.) (*B*) TEM of crude MV preparation (red arrows) from filtered and centrifuged SEZ culture supernatants (scale bar, 200 nm.) (*C*) Immunoblot of crude MVs prepared from SEZ culture supernatants with SzM mAb, the arrow shows SzM. (*D*) Detection of the SzM in different MV fractions by immunoblot with anti-SzM mAb. (*E*) Purified MVs in fraction 2 from 3 independent biological replicates were analyzed by LC/MS. The top 20 proteins ranked by average iBAQ intensity are listed. (*F*) Immunoelectron microscopy detection of SzM in purified MVs with anti-SzM mAb and anti-mouse colloid gold-conjugated secondary antibody. The colloid gold attached to MVs are indicated by red arrows, while the colloid gold in the background are indicated by black arrows (scale bar, 100 nm.) (*G*) MVs purified from the indicated strains were incubated with beads coated with anti-SzM mAb. The beads were visualized with green fluorescent lipophilic PKH67 dye (green) with fluorescence microscopy. (scale bar, 20 μm.)

We next addressed how SEZ could deliver SzM to host cells. MVs shed by gram-positive pathogens can deliver cell wall-anchored bacterial cargo into host cells during infection (25), but MV-mediated delivery of M family proteins to host cells has not been reported. Membrane bound vesicular structures attached to SEZ were detected in electron micrographs (Fig. 3*A*). TEM (Transmission electron microscopy) showed that there were numerous MV-like structures of varying sizes in a crude preparation of vesicles (Fig. 3*B*) and nanoparticle tracking analysis (NTA) revealed that the diameter of the isolated particles varied from 100 nm to more than 400 nm (*SI Appendix*, Fig. S3*A*). Immunoblotting the crude MV sample with an anti-SzM monoclonal antibody revealed SzM (Fig. 3*C*), suggesting that SzM can be incorporated into MVs.

Additional purification of the crude MVs with Optiprep density gradient centrifugation (33) was undertaken to analyze the protein content of SEZ MVs. TEM showed that the purified MVs were found in fractions 2,3, and 4 (*SI Appendix*, Fig. S3*B*), and NTA analysis showed that fraction 2 had a fairly uniform peak centered at ~200-nm diameter (*SI Appendix*, Fig. S3*B*). Additional analyses showed that the proteins associated with fraction 2 were relatively resistant to trypsin treatment (*SI Appendix*, Fig. S3*C*) and similar to other MV-associated proteins (34), TritonX-100 treatment largely ablated the trypsin resistance of these proteins. Together, these observations indicate that fraction 2 vesicles contain proteins associated with lipid vesicles that are in a relatively protease resistance state.

Immunoblotting showed that the SzM protein was only present in samples from fractions 2, 3, and 4 from the Optiprep density gradient that contained purified MVs (Fig. 3*D*). Liquid chromatography–mass spectrometry (LC/MS) was used to identify the proteins in fraction 2. There were 187 proteins that were present in all 3 biological replicates, including SzM (Dataset S1), which was the 7th most abundant protein (Fig. 3*E*). SzM in fraction 2 was completely digested by trypsin after TritonX-100 treatment (*SI Appendix*, Fig. S3*D*). Even without addition of TritonX-100, trypsin treatment reduced the abundance of SzM detected in fraction 2 (*SI Appendix*, Fig. S3*D*), suggesting that a portion of SzM is associated with the outside of SEZ MVs. Immunoelectron microscopy also revealed that at least a proportion of the SzM associated with MVs is accessible to anti-SzM mAb. Colloid gold secondary antibody staining of anti-SzM mAb was observed around the edge of MVs, along with dispersed colloid gold in the background, presumably from lysed MVs (Fig. 3*F*). There was no colloid gold observed either surrounding MVs or in the background in MVs purified from the Δszm mutant (*SI Appendix*, Fig. S3*E*). We also used anti-SzM mAb-coated beads to further demonstrate the association of antibody accessible SzM with SEZ MVs (Fig. 3*G*). MVs bound to anti-SzM antibody-coated beads were detected with the green fluorescent lipophilic PKH67 dye. In this assay, MVs that were purified from WT SEZ bound to the beads and were apparent as a halo after staining with PKH67 dye, whereas no fluorescence was detected when MVs purified from Δszm were used (Fig. 3*G*); MVs purified from CΔszm partially restored the green fluorescent halo (Fig. 3*G*). Together, these data support the idea that SEZ releases MVs harboring abundant SzM that is likely both inside the MVs and in a MV-surface-associated form that is antibody accessible. Thus, SEZ MVs represent a potential vehicle for delivery of SzM to host cells.

### MVs Deliver SzM into hBMECs and Contribute to BBB Disruption.

**Fig. 4.**
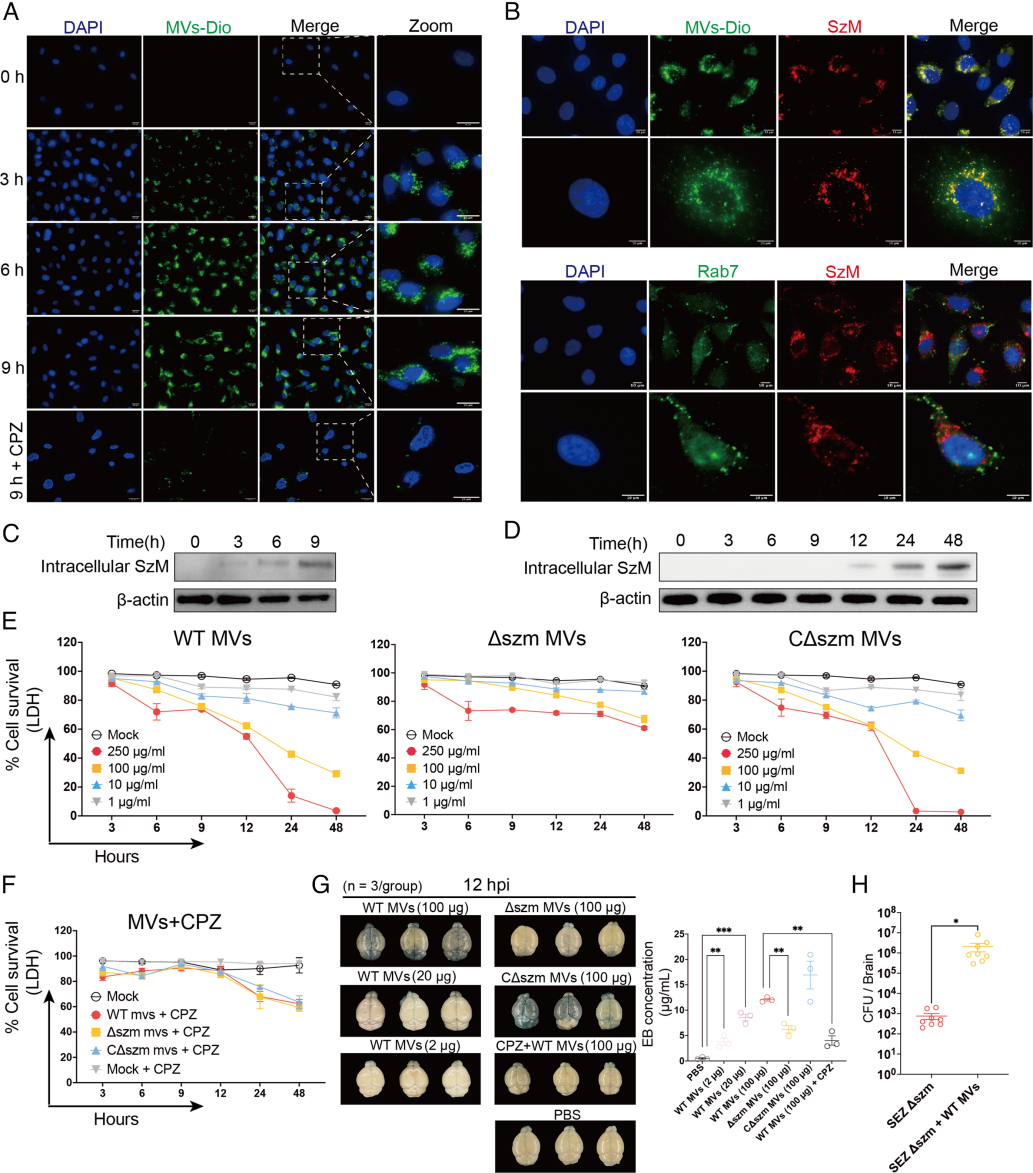
SEZ MVs deliver SzM into hBMECs and contribute to the disruption of BBB integrity. (*A*) Detection of intracellular DiO-labeled (green) MVs in hBMECs with fluorescent microscope at the indicated times after exposure to 500 μg/mL MVs. The endocytosis inhibitor CPZ was added 12 h prior to MV treatment (scale bars, 20 μm). (*B*) Immunofluorescence microscopy observation of colocalization of intracellular MVs/SzM or endosomes/SzM in hBMECs. MVs were labeled with DiO (green), the SzM protein was stained with anti-SzM mAb (red), and endosomes were stained with anti-RAB7 antibody (green). Images were acquired after 9-h exposure to MVs (100 μg/mL) (Bar = 10 μm). (*C* and *D*) Monitoring the amount of intracellular SzM in hBMECs by immunoblot. (*C*) Cells were harvested at 0, 3, 6, and 9 h after treatment with 500 μg/mL SzM-bound MVs (containing ~5.4 μg/mL SzM); (*D*) Cells were harvested at 0, 3, 6, 9, 12, 24, and 48 h after treatment with 10 μg/mL SzM protein; β-actin was used as a reference protein. (*E*) hBMEC survival (by LDH assay) after incubation with increasing concentrations of MVs derived from WT, Δszm, or CΔszm SEZ (n = 3). In the mock group, cells were treated with PBS. (*F*) Protection of hBMECs from cytotoxicity of indicated MVs (250 μg/mL) with the endocytosis inhibitor CPZ (n = 3). (*G*) Evans Blue dye permeability of the BBB in mice 12 h after i.v. injection of MVs purified from the indicated strains at the indicated dosages (200 μL/mouse). CPZ (1 mg/kg) was injected 24 h prior to MV challenge. (** indicates *P* < 0.01 with one-way ANOVA). (*H*) The mice were injected with Δszm (~1 × 10^7^ CFU/mouse) combined with MVs (100 μg/mouse) from WT SEZ through tail vein, then killed at 12 hpi to detect the CFU in the brain. The injection of Δszm with no MV combination was used as negative control (n = 8, * indicates *P* < 0.05 with Student’s *t* test).

SEZ MVs were labeled with 3,3′-dioctadecylox-acarbocyanine perchlorate (DiO), a fluorescent lipophilic dye that has been widely used for tracing the internalization of MVs (35), to test if MV-borne SzM is delivered into hBMECs. MVs were observed inside hBMECs after a 3-h incubation, and with longer incubation times the amount of fluorescent MVs detected within hBMECs increased (Fig. 4*A*). The endocytosis inhibitor CPZ blocked the internalization of MVs into hBMECs, and little fluorescent signal was detected in these cells after 9 h of incubation with MVs (Fig. 4*A*). Notably, at this time point, SzM colocalized with MVs (Fig. 4*B*) rather than endosomes inside hBMECs, suggesting that MV-borne SzM protein does not appear to localize to endosomes, at least after 9 h of MV incubation (Fig. 4*B*). Together, these observations strongly suggest that endocytosis of SEZ MVs provides a mechanism for the delivery of MV-borne SzM into hBMECs.

Comparison of the kinetics of the uptake of SzM into hBMECs from MVs (500 μg/mL MVs containing ~5.4 μg/mL SzM protein, *SI Appendix*, Fig. S4*A*) vs. purified SzM protein (10 μg/mL) revealed that MV-borne SzM protein became detectable inside hBMECs after 3 h and the amount increased at 6 and 9 h after exposure (Fig. 4*C*). In contrast, purified SzM did not become detectable inside hBMECs until 12 h (Fig. 4*D*), suggesting the MVs provide a more efficient means for delivery of SzM into hBMECs than free SzM protein.

To further investigate the contribution of SzM-bound MVs to cytotoxicity, we treated hBMECs with increasing concentrations of MVs derived from WT, Δszm, and CΔszm SEZ (*SI Appendix*, Fig. S4*B*), respectively. As little as 1 μg/mL of SzM-bound MVs (from WT SEZ) induced cytotoxicity in hBMECs after 9-h treatment, and cytotoxicity increased as the concentration of MVs increased (Fig. 4*E*). At a concentration of 250 μg/mL, MVs derived from WT and from CΔszm SEZ killed ~90% of the hBMECs within 24 h, whereas the MVs from the Δszm strain (SzM-free MVs) only killed ~25% of the hBMECs at this point (Fig. 4*E*), indicating that SzM makes a key contribution to SEZ MV-mediated cytotoxicity. The endocytosis inhibitor CPZ reduced the cytotoxicity of SzM-bound MVs to hBMECs (Fig. 4 *E* and *F*) suggesting that endocytosis is required for MV-mediated delivery of SzM to hBMECs.

SzM-bound MVs also increased BBB permeability. In mice, injection of 100 μg MVs derived from WT or CΔszm SEZ led to far more EB dye traversing through the BBB into the brain than mice injected with MVs from Δszm (Fig. 4*G*). This SzM-bound MVs induced BBB permeability showed dosage-dependency, with increasing BBB permeability as doses of MVs were increased (Fig. 4*G*). Furthermore, CPZ protected the BBB integrity in mice injected with WT MVs (Fig. 4*G*). When mice were treated with MVs from WT SEZ combined with the Δszm mutant, there was a significant (*P* = 0.042) increase in the number of Δszm mutant CFU recovered from the brain at 12 hpi (Fig. 4*H*). Together, these data suggest that endothelial cell endocytosis of SzM-bound MVs can largely account for SEZ MV-mediated disruption of the BBB.

### CRISPR/Cas9 Screen Identifies Host Autophagy Pathway Factors Required for SzM Cytotoxicity.

**Fig. 5.**
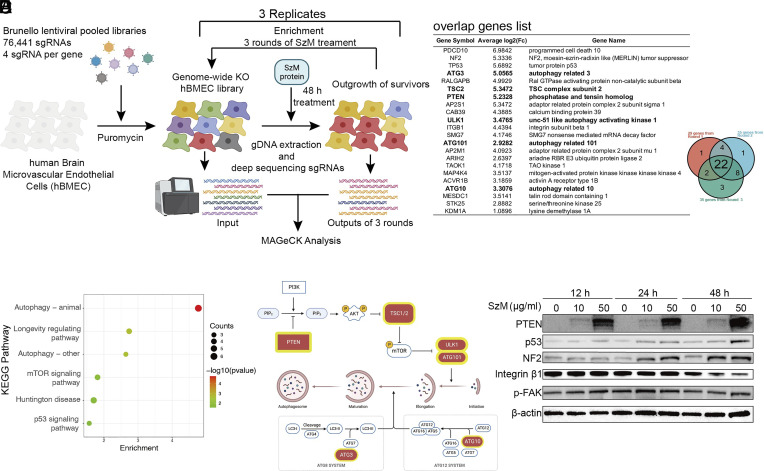
CRISPR/Cas9 screen identifies PTEN-related autophagy pathway factors in susceptibility of hBMECs to SzM-mediated cytotoxicity. (*A*) Schematic of CRISPR/Cas9 SzM protein cytotoxicity screen in hBMECs. Created with BioRender.com. (*B*) Venn diagram of enriched genes from 3 rounds of selection in 3 biological replicates. The enriched 22 overlap genes are listed. The bolded genes belonged to the autophagy pathway. (*C*) KEGG enrichment of the 22 overlap genes. (*D*) Overview of PTEN/Akt/mTOR and autophagy pathway, the enriched genes from the screen are marked with red background and yellow edge. Created with BioRender.com. (*E*) Immunoblot detection of PTEN, p53, NF2, Integrin β1, phosphorylated FAK (p-FAK) in hBMECs after indicated incubation times with different concentrations of the SzM protein (β-actin was used as reference).

A CRISPR screen was carried out to identify hBMEC genes that facilitate SzM-mediated cytotoxicity. The human CRISPR Brunello pooled lentiviral library, which contains four single-guide RNAs (sgRNAs) targeting each protein-coding gene (36) was used to construct a genome-scale hBMEC CRISPR libraries. Purified SzM was used to carry out a screen in 3 replicates of the library (marked as Replicate_A, B, and C). SzM was added to the libraries for 48 h, and in the first round of selection there was 30 to 40% cell death. Survivor cells were outgrown for the next round of selection, and the process was repeated for a total of 3 rounds (Fig. 5*A*). The sgRNAs in the hBMEC CRISPR libraries were sequenced after each round of selection, and the MAGeCK analytic pipeline (37) was used to identify guides that were enriched after each round of selection (*SI Appendix*, Fig. S5*A* and Dataset S2). There was a strong concordance among the 3 replicates (*SI Appendix*, Fig. S5*B*), and sgRNAs targeting autophagy pathway-related genes were overrepresented in all three replicates (*SI Appendix*, Fig. S5*C* and Dataset S3). Twenty-two enriched target genes were common to the 3 rounds of the screen. The most enriched KEGG pathway among these 22 genes was autophagy, which was linked to 6 out of the 22 genes (Fig. 5 *B* and *C*). The longevity regulating and mTOR signaling pathways were at a lower rank but potentially relevant to autophagy as well (Fig. 5*C*).

The integrin was on the top output genes of CRISPR screen and it was known as an important target for GAS and GBS infection, leading to BBB disruption and autophagy (38–40). We found that the Integrin β1 was important for hBMEC endocytosis of MVs, as an anti-integrin β1 antibody significantly blocked the entrance of MVs into cells (*SI Appendix*, Fig. S6*A*). However, the SzM protein itself was likely not involved in endocytosis process, because the SzM-free MVs and MVs from WT strain had similar cell entry efficiency (*SI Appendix*, Fig. S6*A*). Moreover, SzM protein did not induce significant integrin β1 variation within 24-h treatment (Fig. 5*E*), and we did not identify interactions between SzM and integrin β1 (*SI Appendix*, Fig. S6*B*). Notably, the phosphorylation level of FAK, which is a downstream of integrin in the integrin/FAK/PI3K/Akt signal pathway (41), was not increased (Fig. 5*E*), suggesting that at least in our hBMEC model, integrin β1 was highly unlikely involved in signal transduction from the SzM but only relevant to endocytosis process.

The autophagy-linked genes also included NF2 (Neurofibromatosis type 2), TP53 (Tumor Protein p53), and PTEN, which act as upstream regulators of autophagy, the mTOR-autophagy axis regulating complex (TSC1, ULK1, and ATG101), and the autophagosome formation-related proteins ATG3 and ATG10 (Fig. 5*D*). NF2, p53, and PTEN all showed elevation in hBMECs after exposure to SzM protein, but the elevation in PTEN levels were more pronounced than in NF2 and p53, especially after 12 h of SzM treatment (Fig. 5*E*). PTEN has a key role in a variety of biological processes, including autophagy through its regulation of the PI3K/Akt/mTOR pathway (42, 43). SzM-induced PTEN elevation occurred in a dosage-dependent fashion (Fig. 5*E*), and was also observed in THP-1 cells (human leukemia cell line) (*SI Appendix*, Fig. S7). Together, these data strongly suggest that defects in the autophagy pathway, especially PTEN-related autophagy pathway, confer resistance to SzM-mediated cytotoxicity in hBMECs.

### Inhibition of MV-Borne SzM-Induced Autophagy Protects BBB Integrity.

**Fig. 6.**
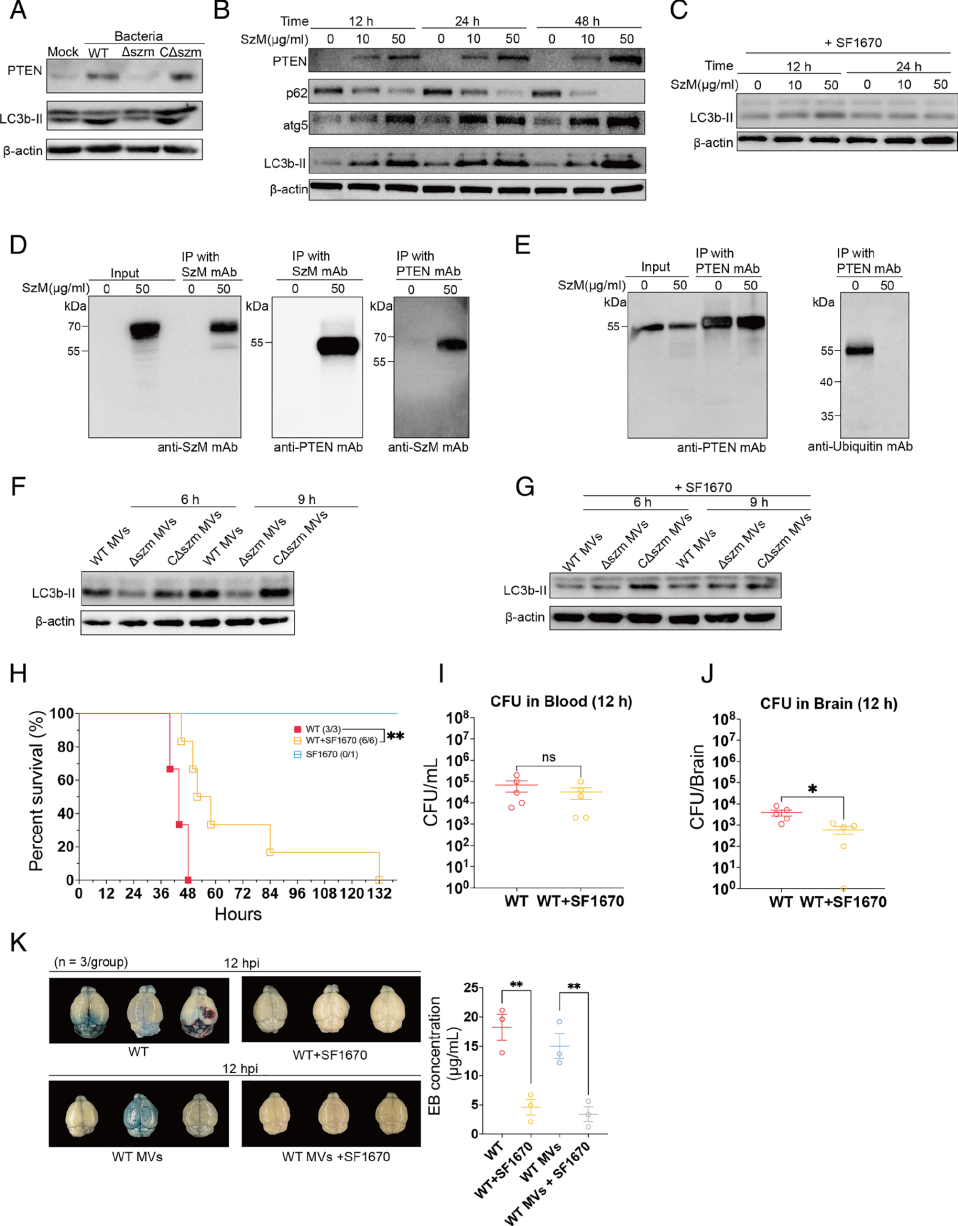
The MV-borne SzM-induced PTEN elevation-dependent autophagic cell death and BBB permeability increase can be impeded by inhibition of PTEN activity. (*A*) Immunoblot detection of PTEN and LC3b-II in hBMECs 3 hpi with the indicated SEZ bacterial strain at MOI = 1:10 (β-actin was used as reference in all immunoblot detections). (*B*) Immunoblot detection of p62, ATG5, PTEN, and LC3b-II in hBMECs 12, 24, and 48 h after incubation with the indicated amounts of SzM protein. (*C*) Immunoblot detection of LC3b-II in hBMECs treated with PTEN activity inhibitor SF1670 12 and 24 h after incubation with the indicated amounts of SzM protein. (*D*) Immunoblot detection of the interaction of SzM and PTEN. Input was the SzM treated (PBS in control) hBMECs lysis (12 h). The Co-IP was conducted with anti-SzM antibody, and the immunoblot was detected with anti-PTEN antibody. The reverse was also done using anti-PTEN antibody for Co-IP and anti-SzM antibody for immunoblot. (*E*) The same input sample in *D* was used to Co-IP with anti-PTEN antibody. Then washing the precipitated PTEN protein off and detecting its ubiquitylation with anti-Ubiquitin antibody. (*F* and *G*) Immunoblot detection of LC3b-II in hBMECs after treatment with MVs (100 μg/mL) derived from WT, Δszm, or CΔszm SEZ strains for indicated times without (*F*) or with (*G*) SF1670. (*H*) Survival curves of C57BL/6 mice with or without pre-injection of SF1670 at 3 mg/kg 24 h prior to WT SEZ bacterial challenge. The SF1670 alone group was used to evaluate the lethality of this drug with only one mouse (** indicates *P* < 0.01 and ns indicates no significant difference with the log-rank test vs. WT). (*I* and *J*) Bacterial burden in the WT SEZ challenged mice blood (*I*) and brain (*J*) at 12 hpi, or combined with SF1670 injection 24 h prior to bacterial challenge (* indicates *P* < 0.05 and ns indicates no significant difference with Student’s *t* test vs. WT); (*K*) Permeability of the BBB in mice at 12 h post WT SEZ or WT MVs without or with SF1670 injection 24 h prior to SEZ or MVs challenge evaluated with the Evans Blue dye assay (** indicates *P* < 0.01 with Student’s *t* test).

Initially, hBMECs were used to explore if SEZ infection triggers PTEN induction of autophagy. PTEN and LC3b (microtubule-associated protein 1 light chain 3B), a marker of the activation of autophagy and the formation of autophagosomes (44, 45), were monitored after hBMECs were infected with WT, Δszm, or CΔszm SEZ strains. Three hours after infection, the levels of PTEN and LC3b-II were both elevated in cells infected with WT and CΔszm SEZ but not with the Δszm strain (Fig. 6*A*). Elevation of PTEN levels 12 h after treatment of hBMECs with SzM was also observed and associated with increased levels of LC3b-II (Fig. 6*B*). There was also an elevation of ATG5 (a key protein involved in the extension of the phagophoric membrane in autophagic vesicles) in hBMECs (Fig. 6*B*). Meanwhile, the abundance of p62, a known autophagic substrate, decreased in hBMECs after SzM protein treatment (Fig. 6*B*). By blocking autophagosome–lysosome fusion with Bafilomycin A1, the accumulation of LC3b-II was more apparent in hBMECs after exposure to the SzM protein (*SI Appendix*, Fig. S8*A*). To monitor SzM-stimulated autophagic flux in hBMECs, a red fluorescent protein (RFP)–GFP–LC3b fusion protein was used. With this construct, when autophagosomes fuse with lysosomes, GFP intensity is reduced due to acidic conditions, while the RFP signal intensity remains relatively stable (46). The autophagic flux was evaluated by counting the red foci (autolysosome) and the yellow foci (autophagosome). After incubation of hBMECs with SzM for 12 h, the number of autophagosomes increased, and the GFP and RFP signal intensities were similar and colocalized, consistent with the idea that SzM stimulates the formation of autophagosomes and activates autophagy. At 24 h, the GFP signal intensity was reduced whereas the RFP intensity was little changed, suggesting that autolysosomes, resulting from the fusion of autophagosomes and lysosomes had formed by this point (*SI Appendix*, Fig. S8*B*). These observations suggest that SzM triggers autophagy, a process that has been linked to autophagy-dependent cell death (47, 48).

Addition of the PTEN inhibitor SF1670 (49) to hBMECs nearly abolished SzM stimulation of autophagy activation, and little change in the amounts of LC3b-II in hBMECs were observed 12 and 24 h after SzM treatment (Fig. 6*C*). These observations are consistent with the idea that SzM triggers PTEN-related autophagy and are consistent with the results of the CRISPR screen. Mechanistically, using coimmunoprecipitation we found that SzM interacts with PTEN (Fig. 6*D*). Moreover, the ubiquitylation level of PTEN was reduced after exposure to SzM, suggesting that the intracellular SzM blocks PTEN degradation by inhibiting its ubiquitylation (Fig. 6*E*). Furthermore, SF1670 and the autophagy inhibitor 3-MA (inhibitor of PI3K) reduced the cytotoxicity of SzM to hBMECs (*SI Appendix*, Fig. S9*A*), and *pten* KO hBMECs were more resistant to SzM-mediated cytotoxicity compared to wild-type hBMECs (*SI Appendix*, Fig. S9 *B* and *C*). Neither SF1670 nor pten KO influenced the endocytosis of MVs by hBMECs (*SI Appendix*, Fig. S9*D*). These results strengthen the link between SzM cytotoxicity and PTEN-dependent autophagic cell death.

MV-borne SzM also induced autophagy in hBMECs. When hBMECs were treated with MVs derived from WT, Δszm, and CΔszm SEZ, the abundance of LC3b-II was lower in cells treated with MVs derived from Δszm than the other two strains (Fig. 6*F*). In addition, SF1670 treatment largely blocked the generation of LC3b-II in cells treated with MVs from all three strains (Fig. 6*G*). These observations suggest MVs from WT SEZ can stimulate PTEN-dependent autophagy in hBMECs, and that MV-borne SzM plays an important role in this process.

Notably, in mice, injection of SF1670 prior to SEZ challenge significantly (*P* = 0.0073) extended the survival time and delayed the time when all mice died from 48 to 132 h (Fig. 6*H*). Despite these marked differences in clinical outcomes, the SEZ burden in blood in SF1670-treated and -untreated animals at 12 hpi was similar (Fig. 6*I*); however, the bacterial burden in brain was significantly reduced by SF1670 treatment (Fig. 6*J*). Finally, SF1670 pretreatment reduced the amount of EB dye in the brain 12 h after challenge with SEZ or SEZ-derived MVs (Fig. 6*K*). Together, these data strongly suggest the MV-borne SzM-induced BBB disruption during SEZ infection is at least partially dependent on PTEN activation triggering autophagic cell death.

## Discussion

Our findings show that the hypervirulent SEZ ST194, a group C Streptococcus, releases MVs containing the M protein SzM. These SzM-laden lipid vesicles are sufficient to disrupt the murine BBB by increasing BBB permeability and function as a vehicle for SzM delivery into endothelial cells, where it causes cytotoxicity. Inhibition of endocytosis blocked MV-borne SzM-induced cytotoxicity to hBMECs and blocked the increase in permeability of the murine BBB and SEZ penetration into the brain. Our CRISPR screen strongly suggested that SzM causes cytotoxicity in hBMECs by stimulating PTEN-related autophagic death. Moreover, in a mouse model, inhibition of PTEN activation nearly abolished SEZ or SEZ-derived MV disruption of the integrity of the BBB. Collectively, our findings reveal that SzM-containing MVs play a key role in SEZ pathogenicity and suggest avenues for therapeutics.

Streptococcal M proteins have been investigated for many years and have been linked to pathogenicity (20). M proteins are linked to the bacterial cell surface by their covalent attachment to peptidoglycan. Phenotypes attributable to MV-borne M proteins have not been reported previously. However, phenotypes associated with free M proteins are known. Neutrophil-derived granule proteases were reported to cleave the M protein from the cell surface, and it was also shed naturally from the GAS surface (50, 51). The released M protein had distinct functions, including triggering vascular leakage and tissue injury (51), and stimulating caspase-1-dependent NLRP3 inflammasome activation (52, 53), not associated with cell surface–associated M protein. Thus, M and M-like proteins, such as SzM, may carry out different functions depending on whether or not they are attached to the streptococcal cell surface. Given our findings presented here, it seems likely that most or all M proteins can become associated with streptococcal MVs and determining the functions of MV-borne M proteins should be a fruitful avenue of research. In general, MVs provide an ideal platform for delivery of bacterial cell surface proteins, such as M proteins, to host cells.

MVs from other grampositive bacteria have been shown to harbor cytotoxic factors and to be an efficient means of delivery of such factors to host cells. For example, the intact *Staphylococcus aureus* MVs are more cytotoxic than lysed MVs (54, 55), and MVs bearing the *Mycobacterium ulcerans* mycolactone toxin are more cytotoxic than purified toxin alone (56). Our findings also suggest streptococcal MV-borne SzM has greater cytotoxic efficiency to hBMECs than purified SzM (Figs. 2*B* and 4*E*), suggesting that MV-borne SzM can enter host cells more efficiently than free SzM. Endocytic uptake of MVs, which is known as an important route for MV uptake by host cells (33, 57), likely explains how MV-borne SzM enters hBMECs. The integrin, which is on the top rank of our CRISPR screen outcomes, probably participated in this MV endocytosis process of hBMECs, this molecule had been identified in mediating extracellular vesicles uptake in other cell types as well (58). Within the hBMEC cytoplasm, we observed colocalization of SzM and MVs, suggesting that SzM may be partially exposed on the MV surface if it directly modulates host cell processes that trigger PTEN-dependent autophagy. Consistent with this possibility, we found that MV-borne SzM is accessible to antibodies and somewhat sensitive to protease degradation independent of Triton-X treatment.

Although we identified the SzM could induce PTEN elevation by blocking its ubiquitylation, and subsequently lead to PTEN-dependent autophagic cell death, there could be another cell death pathway attributed to the SzM cytotoxicity as well. Besides PTEN and several autophagy-relevant genes endorsed by KEGG analysis, the CRISPR screen top outcomes also included PDCD10 (Programmed cell death 10), p53 and NF2 that associated with apoptosis and autophagy (59–62). Considering that the SF1670 and *pten* KO were not capable to completely rescue hBMECs from SzM cytotoxicity, the MV-borne SzM-induced cell death pathway could be more complex. Nevertheless, our findings suggest PTEN-induced autophagic cell death is an indispensable part of explanation for SzM cytotoxicity.

MVs have been described in several streptococcal species and they are thought to contribute to pathogenicity through disrupting host barriers and enabling pathogen invasion and dissemination (25, 63). Physical barriers, such as the BBB, play vital roles in preventing pathogens from invading important organs, like the brain (15). We found that MV-borne SzM could disrupt the murine BBB via MV delivery. Inhibition of endocytosis or PTEN activation both protected BBB integrity, suggesting that the MV delivery of SzM and subsequent PTEN stimulation of autophagic endothelial cell death are both critical for the BBB disruption required for SEZ invasion of the CNS. Taken together, our findings are consistent with a model in which SEZ-derived MVs laden with SzM trigger PTEN-dependent autophagic death in brain endothelial cell, enabling SEZ to enter the CNS via a paracellular pathway (*SI Appendix*, Fig. S11), consistent with our previous observations (18). Group B Streptococci also hijack autophagy to penetrate the BBB, but this pathogen traverses the BBB via a transcellular pathway (64).

This study deepens understanding of the mechanisms that SEZ relies on to invade the CNS. Furthermore, our data suggest that inhibition of the endocytosis of MVs or of PTEN-triggered autophagic cell death represents potential therapeutic avenues to prevent SEZ meningitis.

## Materials and Methods

### Bacterial Strains and Growth.

SEZ ATCC35246 was cultured in THB medium or on THB agar plates at 37 °C. *E. coli* DH5α and *E. coli* BL21 (DE3) were grown at 37 °C in the Luria Broth medium or on agar plates. The following concentrations of antibiotics were added as needed: ampicillin (Amp, 100 μg/mL), spectinomycin (Spc, 50 μg/mL), and kanamycin (Kan, 100 μg/mL). Further details regarding bacterial mutant generation, growth curve detection, MV isolation and identification, and animal infection experiments are provided in *SI Appendix, Materials and Methods*.

### Cell Culture.

hBMECs were purchased from ScienCell Research Laboratories (Catalog #1000). HEK293T cells (ATCC® CRL-3216TM) and THP-1 cells (ATCC® TIB-202™) were purchased from American Type Culture Collection. The hBMECs and HEK293T cells were cultured in Dulbecco’s modified Eagle’s medium (Gibco, No. c1995500bt) supplemented with 10% fetal bovine serum (Gibco, No.10091148); the THP-1 cells were cultured in RPMI 1640 (Gibco, No. c11875500bt) medium with 10% fetal bovine serum (Gibco, No.10091148). To differentiate THP-1 cells into MΦs, cells were stimulated with 5 ng/mL phorbol 12-myristate 13-acetate (PMA, Sigma Aldrich, 79346-1MG) in RPMI complete medium for 72 h and rested in PMA-free RPMI-1640 medium without FBS for an additional 48 h. Further details regarding cell survival assay, microscopy observation, construction of derivative hBMECs, protocol of inhibitor usage, CRISPR screen, and endothelium monolayer model construction assay are provided in *SI Appendix, Materials and Methods*.

## Supplementary Material

Appendix 01 (PDF)

Dataset S01 (XLSX)

Dataset S02 (XLSX)

Dataset S03 (XLSX)

Dataset S04 (XLSX)

Movie S1. Observation of hBMECs expressing SzM-GFP fused protein with live-cell imaging (scale bar, 10μm).

Movie S2. Observation of hBMECs expressing GFP protein with live-cell imaging (scale bar, 10μm).

## Data Availability

The CRISPR Screen sequencing raw data have been deposited to GEO database with Series GSE217997 (65). The LC/MS raw data have been deposited to Mendeley Data (66). All other study data are included in the article and/or *SI Appendix*.
