# Supplementary material for: Membrane vesicle delivery of a streptococcal M protein disrupts the blood–brain barrier by inducing autophagic endothelial cell death
Source: Proc Natl Acad Sci U S A. 2023 Jun 5;120(24):e2219435120. doi: 10.1073/pnas.2219435120 (PMC10268326; doi:10.1073/pnas.2219435120)
Supplement: Supplementary file 1 — Appendix 01 (PDF) [file pnas.2219435120.sapp.pdf]

PNAS

Supporting Information for

Membrane vesicle delivery of a streptococcal M protein disrupts the blood-brain barrier by inducing autophagic endothelial cell death

Fei Pan[a], Mingli Zhu[a], Ying Liang[a], Chen Yuan[a], Yu Zhang[a], Yuchang Wang[a], Hongjie Fan[ab], Matthew K. Waldor[c,d,e] and Zhe Ma[ab]*

Corresponding author: Zhe Ma
Email: mazhe@njau.edu.cn

This PDF file includes:

- SI Materials and Methods
- Figures S1 to S11
- Legends for Movies S1 to S2
- Legends for Datasets S1 to S4
- Table S1
- SI References

Other supporting materials for this manuscript include the following:

- Movies S1 to S2
- Datasets S1 to S4

Materials and Methods

Ethics statement.

All animal experiments were performed with protocols approved by the Laboratory Animal Welfare and Ethics Committee of Nanjing Agricultural University in accordance with the Laboratory Animal Guideline for ethical review of animal welfare (GB/T 35892-2018).

Bacterial Strain construction.

Single *szm* gene deletions in SEZ ATCC35246 were constructed as previously described based on the temperature-sensitive shuttle plasmid pSET4s (1, 2). Using SEZ ATCC35246 genomic DNA as a template, fragments upstream and downstream of *szm* were amplified by PCR with primers szm_up_F/szm_up_R and szm_down_F/szm_down_R respectively, and then inserted into pSET4s to construct the pSET4s-del-szm recombinant plasmid for *szm* deletion. The silent mutant *szm* allele was amplified with a synonymous a to g mutation at *szm* bp 864 (Figure S1A), which was then inserted into pSET4s derivative with the *szm* upstream (amplified with primers Cszm up_F/Cszm up_R) and downstream (amplified with primers Cszm_down_F/Cszm_down_R) fragments to construct the pSET4s-comp-szm plasmid for in-situ complementation (CΔszm) in the Δszm background. Plasmids were introduced into competent SEZ cells via electroporation (Bio-Rad, Gene Pulser Xcell, Voltage: 2300 V, Capacitance: 25 μF, Resistance: 200 Ω, Cuvette: 1 mm). The transformed bacteria were grown at 37 °C in THY (THB + 2% yeast extract) broth plus spectinomycin (Spc) to generate single-crossover mutants. Double-crossover mutants were generated by repeatedly passaging the single-crossover strains at 28 °C on THY without Spc. Gene deletions were verified by PCR and Sanger sequencing (GENEWIZ).

PCR was routinely performed using Phusion high-fidelity DNA polymerase (NEB, M0530S), and plasmids were constructed via Gibson assembly using the NEBuilder HiFi DNA assembly master mix (NEB, E2621L). Primer sequences are listed in Table S1 in the supplemental material.

Growth curve.

To monitor the growth of SEZ strains in Todd Hewitt broth (THB) medium, bacteria in logarithmic growth phase were diluted 1:100 into fresh THB at 37 °C, 180 rpm. The optical densities at 600 nm (OD_{600}) of the cultures was monitored hourly using a biospectrometer (Eppendorf). For the growth of SEZ strains in porcine cerebrospinal fluid (CSF), the fresh porcine CSF of SPF Bamaxiang pigs was donated by Jiangsu Academy of Agricultural

Science (JAAS). Bacteria in logarithmic growth phase were diluted 1:100 into fresh CSF at 37 °C, 180 rpm. The bacterial culture in CSF were sampled at 0, 3, 6, 9, 12 h, then 10 μl of each sample was serially diluted and spread on THB agar plates for CFU counting after overnight culture at 37 °C.

Animal experiments.

Six-week-old female C57BL/6J mice, purchased from GemPharmatech Co., Ltd., were used for all animal experiments. Mice were intravenously injected with 5 × 10^6 CFU of wild-type (WT) SEZ and CΔszm SEZ, or 1 × 10^7 CFU SEZ Δszm mutant via the tail vein in Fig. 1A-D and Fig. 6H-K. The bacteria were resuspended in phosphate-buffered saline (PBS, pH = 7.4), and the control group was injected with the same volume of PBS alone. To determine brain burdens, the mice were deeply anesthetized using isoflurane and cervically dislocated at 3, 6, 12, and 24 h after injection, then the brain was homogenized, and serial dilutions of the homogenates were plated on THB agar plates to enumerate bacterial CFU. For the bacterial burden in the blood, 10 μl of blood was drawn and serial dilutions were plated on THB agar plates. In Figure 4H, mice were individually injected via the tail vein with 10^7 CFU of SEZ Δszm strain or the same amount of SEZ Δszm strain combined with WT MVs (final concentration: 500 μg/ml) at a volume of 200 μl/mouse. The procedures used to determine brain burdens were the same as those described above.

Cell survival assay.

The hBMECs or hBMEC *pten* KO cells were treated with different concentrations of endotoxin-free SzM protein. Following treatment, at each time point assayed, surviving cells were quantified either by trypan blue exclusion (0.4% trypan blue) and counted on a Countess II Automated Cell Counter (Thermo Fisher Scientific), or with an LDH Cytotoxicity Assay Kit (Beyotime, c0017) for quantifying the total LDH in surviving cells. For the LDH assay, all surviving cells after treatment were centrifugated at 1000 rpm. The LDH release solution was used to release LDH in cells at 37 °C for 1 h. The LDH was measured by the LDH Cytotoxicity Assay Kit on the Infinite 200 PRO microplate plate reader (TECAN).

Evans Blue analysis.

Evans Blue (EB) leakage was used to assess BBB permeability as described (3). Mice were injected with SEZ or MVs (200 μl/mouse); the EB (2%, 100 μl/mouse) was injected in the tail vein 0.5 h before perfusion. Deeply anesthetized mice were perfused with 0.9% saline containing 10 U/ml heparin (10 ml) via the cardiac vein. Harvested brains were

dissected and photographed. Formamide was used to extract the EB out of the brain tissue. The EB amounts were determined as absorbance at OD_{620}.

Construction of derivative hBMECs with lentivirus

An hBMEC cell line stably expressing the RFP-GFP-LC3b protein was constructed as follows. The vectors psPAX2 and pMD2.G were gifts from Didier Trono (Addgene plasmid # 12260, # 12259). The vector pLVX-Puro-RFP-GFP-hLC3B was provided by Yongjie Liu's lab at Nanjing Agricultural University. HEK293T cells were transfected with psPAX2, pMD2.G, and pLVX-Puro-RFP-GFP-hLC3B to produce lentivirus. The hBMECs were infected with lentivirus and cultured in the presence of 1 mg/mL puromycin for 7 days. The surviving RFP-GFP-LC3b hBMECs were outgrown and detected by a confocal microscope (Nikon A1).

The vector lentiCRISPRv2 puro was a gift from Brett Stringer (Addgene plasmid # 98290). To knockout the *pten* in hBMEC cell line, firstly, the vector lentiCRISPRv2 puro was digested with BsmBI (NEB, R0739), a pair of annealed oligos (Table S1, pten-ko1-F/R) was cloned into the single guide RNA scaffold. Secondly, lentiCRISPRv2 puro (with sgRNA cloned), psPAX2 and pMD2.G were co-transfected into HEK293T cells to generate lentivirus. Thirdly, the hBMECs were infected with lentivirus and cultured in the presence of 1 mg/mL puromycin for 7 days. The surviving hBMEC *pten* KO cells were outgrown and detected by immunoblot.

Inhibitors.

The endocytosis inhibitors chlorpromazine (CPZ, HY-B0407A) and methyl-β-cyclodextrin (HY-101461), autophagosome-lysosome fusion inhibitor Bafilomycin A1 (BafA1, HY-100558) and PTEN inhibitor SF1670 (HY-15842) were purchased from MedChemExpress and used at final concentrations of 10 nM, 0.38 mM, 100 nM and 100 nM, respectively in vitro. 3-Methyladenine (3-MA) was purchased from Selleck and used at final concentration of 2 mM. For in vivo treatments, mice were pretreated 24 h (i.v) prior to infection with chlorpromazine (1 mg/kg) or SF1670 (3 mg/kg). At these concentrations, these inhibitors did not demonstrate significant cytotoxicity toward hBMECs or mice.

Construction of CRISPR/Cas9 library in hBMEC.

The Human Brunello CRISPR knockout pooled library, created by David Root and John Doench, was obtained from Addgene (#73178); the library contains four sgRNAs targeting each of the annotated human protein-coding genes (19,114 genes). The following steps were used to generate the hBMECs CRISPR library. We aimed to achieve 800 × coverage

(800 cells per perturbation). With 76,441 sgRNAs in the library, this translates into 6 × 10^7 infected cells. To avoid insertions of multiple sgRNAs per cell, 30-50% infection efficiency is desired. We determined the titer of the Brunello lentiviral library on hBMECs to yield 40% infectivity; therefore, a total of 1.6 × 10^8 cells were transduced. Then, a total of 1.6 × 10^8 trypsinized hBMECs (100 ml) were prepared in DMEM with 10% fetal bovine serum (FBS) supplemented with polybrene (8 mg/ml) and Brunello library virus, aliquoted (2 ml/well) among five 12-well plates. Lentiviral transduction was performed using spin infection conditions for 2 h at 2,000 rpm. Plates were then incubated at 37 °C with 5% CO_2 for 8 h, followed by seeding of 5 × 10^6 trypsinized cells in each of 21 T225 flasks. After 24 h of incubation, media were replaced with DMEM (10% FBS) supplemented with puromycin (1 mg/ml). Following an additional 4 days of selection, transduced cells were trypsinized, counted, and cryopreserved with 1 × 10^7 cells/ml.

Positive selection screen using the hBMEC CRISPR Brunello Libraries.

The hBMEC CRISPR/Cas9 library was seeded in eight T225 flasks with 2 × 10^7 cells each and incubated for 3 days. The hBMECs doubled every 3 days, generating a total of 3.2 × 10^8 cells, which were divided into four parts, yielding 8 × 10^7 cells per experimental condition (~1,000× coverage per perturbation in each library). A quarter of the cells were used the input library and the remaining three quarters for SzM cytotoxicity screens. The rest cells were divided into 3 aliquots for 3 screening replicates. For each screening, the cell libraries were treated with purified SzM protein diluted in DMEM (10% FBS) at a final concentration of 50 μg/ml for 48 h; when ~50% of cells were dead, the surviving cells were out grown in fresh DMEM (10% FBS) without SzM protein. These cells were divided into two parts, one part for genomic DNA extraction, and the other for the next round of screening. The same treatment with 50 μg/ml purified SzM protein for 48 h was used in the 2nd round and the 3rd rounds, using the same protocol outlined above. After the 3rd round, all cells were harvested for genomic DNA extraction.

Genomic DNA preparation and analyses of screen results.

Genomic DNA was obtained from the input library and after each of the three rounds of screening with the Blood and Cell Culture DNA Maxi Kit from QIAGEN (No.13362). PCR was used to amplify sgRNA sequences as described (4), and the products were sequenced for amplicon sequencing analysis by the Novegene Company. The sequencing results of CRISPR/Cas9 screening data were processed and analyzed using the MAGeCK and MAGeCK-VISPR algorithms as described (5). Dot plots of enriched KEGG pathways were plotted using the clusterProfiler package (6).

Isolation and identification of SEZ membrane vesicles.

Streptococcus equi subsp. *zooepidemicus* (SEZ) ATCC35246 and its derivatives (Δszm and CΔszm) were cultured in THB with shaking to an OD_{600} of 1.5 ± 0.1. Hyaluronidase (Sigma, No.H3506) was used to hydrolyze hyaluronic acid in the bacterial solution, making the cells easier to disassociate. The bacterial culture supernatant was filtered through a 0.22 μm filter and concentrated 20-fold with a 100-kDa tangential flow filtration system (Pall, OA100C12). The retentate was subjected to ultracentrifugation at 150,000 × g for 3 h at 4 °C to pellet the vesicles and leave soluble proteins in the supernatant. For the purification of MVs, the MVs pellet was suspended in 40% Optiprep density gradient medium (Sigma, No. D1556) and overlaid with gradient layers of Optiprep in a range of 35%, 30%, 25%, 20%, 15%, and 10%. After ultracentrifugation at 150,000 × g for 16 h at 4 °C, 2 ml of each fraction was subjected to SDS-PAGE and stained with a silver stain kit (Thermo Scientific, No.24612). The same fraction was pooled and the Optiprep medium was removed with PBS using a 100 kDa Amicon® Ultra 15 mL Centrifugal Filters (Millipore, UFC910096). The MVs protein concentration was determined by Bradford assay (Thermo Scientific, No.23200). Nanoparticle tracking analysis was performed to assess the number and size of purified MV particles suspended in 2 ml PBS with Nanosight NS300 Sub Micron Particle Imaging System (Malvern).

MVs tryptic digestion assay.

Purified MVs were incubated with 0.04% trypsin (Gibco, No. 27250018) for 10, 20, and 30 min after 10 min with or without pretreatment with 0.1% TritonX-100 at 37 °C. The reaction was stopped with 5 × protein loading buffer. The sample analyzed in sliver-stained SDS-PAGE and by immunoblotting using an anti-SzM monoclonal antibody (mAb) (1:1000) as described (7).

Proteomic analysis of MVs by MS and LC/MS.

For identification of the protein components of purified MVs in fraction 2, samples were prepared for Liquid chromatography–mass spectrometry (LC/MS) analysis using filter-aided sample preparation. The nano-liter flow rate HPLC liquid system, System EASY nLC1000, was used for separation. The peptides were separated by chromatography and analyzed by mass spectrometry using a Q-Exactive mass spectrometer (Thermo Scientific) with the following parameters: analysis time: 120 min; detection mode: positive ion; parent ion scanning scope: 300-1800 m/z. The raw database was analyzed using MaxQuant 1.6.10 to perform a spectrum match of peptides or proteins in the UniProt database:

P20210100146_zijianku_20210207.fasta. Gene ontology analysis was performed utilizing the ClusterProfiler R package.

Transmission electron microscopy (TEM).

For imaging SEZ wild-type ultrathin sections, the bacteria were cultured to an OD_{600} of 1.5 ± 0.1, collected by centrifugation, and fixed with 4% paraformaldehyde and 8% glutaraldehyde. For the negative staining TEM, the MVs were fixed with 2% Paraformaldehyde (PFA) for 5 min. MVs were loaded on the grids, incubated for 5 min and then immediately stained with 2% uranyl acetate (UA) on the surface of the TEM grids. Samples were sent to the Electron Microscope Core of Nanjing Agricultural University for further treatment and observation with the TEM (HITACHI, HT7800).

For immunoelectron microscopy of MVs, samples were fixed with PFA at a final concentration of 2% (v/v) for 5 min. The MVs were loaded on the grids and incubated for 10 min, rinsed with PBS 3 times, 10 min each. The grids were treated with 0.05 M glycine for 10 min to quench free aldehyde groups, then rinsed with PBS. Next, grids were transferred to a drop of blocking buffer (PBS containing 1% BSA) for 30 min at room temperature and then incubated overnight at 4 °C with anti-SzM mAb, diluted at 1:20 in PBS with 0.1% BSA. After washing grids with five separate drops (50 μL) of PBS containing 0.1% BSA for 10 min each, the grids were transferred to a drop of 10 nm gold particle conjugated anti-mouse IgG antibody (Abcam, ab39619), diluted at 1:100 in PBS containing 0.1% BSA. Grids were then washed with five separate drops (50 μL) of PBS containing 0.1% BSA for 10 min each and two separate drops (50 μL) of distilled water. The grids were submitted to perform negative staining and imaged with TEM in the Electron Microscope Core of Nanjing Agricultural University (HITACHI, HT7800).

MVs immunoprecipitation assay.

MVs derived from WT, Δszm or CΔszm were incubated with anti-SzM mAb overnight at 4 °C, and then the mixture was incubated with protein G agarose for 2 h at 4 °C. The beads were collected by centrifugation, washed with chilled PBS, and then the vesicles were stained by PKH67 (Sigma, p7333) at a final concentration of 1 μM for 30 min at 37 °C. The beads were then loaded on slides and images were obtained on a fluorescence microscope (Zeiss, Axio observer).

Tissue culture endothelium monolayer model construction and assay.

Transwells were purchased from CORNING (No.3470), and hBMECs were seeded on the apical side of collagen-coated polytetrafluoroethylene 0.4 μm pore-size membranes

(Corning, No.3470) for the barrier integrity assay. Cells were grown for 3 days to form intact monolayers. The 10^6 CFU of WT SEZ, Δszm SEZ, and CΔszm SEZ were added to the upper chamber of the transwells containing hBMEC monolayers and incubated at 37 °C in 5% CO_2 for 6 h. The TEER values were measured by Millicell® ERS-2 (Millipore) every hour. For SzM protein treatment, SzM protein at a concentration of 10 or 50 μg/ml was applied to the tissue culture endothelium monolayer model for 12 and 24 h. TEER values were measured after the addition of SzM protein at 12 and 24 h.

Protein expression and purification.
For bacterial expression of SzM, the *szm* gene was PCR amplified from SEZ with primers szm_truncated_F/szm_truncated_R, and subcloned into pET-28a, to yield pET-28a-szm plasmid. The recombinant plasmid was expressed in *E. coli* BL21 (DE3). Bacteria were grown in LB containing kanamycin at 37 °C until OD_{600}=0.6~0.8, then induced with 1 mM isopropyl β-d-1-thiogalactopyranoside and grown for a further 5 h at 37 °C. Bacteria were collected by centrifugation and resuspended in lysis buffer (300 mM NaCl, 100 mM Tris-HCl, pH=8, 10 mM imidazole) with PMSF (Biosharp, No.BS071B). After ultrasonic lysis, the soluble SzM protein was purified by HisTrap HP 5 mL column (Cytiva, No.17524801) on the ÄKTA system (GE Healthcare). The 10 kDa Amicon® Ultra 15 mL Centrifugal Filters (Millipore, UFC901024) were used to remove imidazole in the purified SzM protein. The endotoxin of purified SzM protein was removed by Pierce High-Capacity Endotoxin Removal Resin (Thermo, No.88276). Endotoxin-free SzM Protein was aliquoted and flash-frozen in liquid N_2 for storage at −80 °C. All the SzM protein used in this study was endotoxin-free SzM protein. The endotoxin-free SzM protein was boiled for 30 min as the heat inactivated (HI) protein treatment control in *SI Appendix*, Fig. S9C.

Live-cell observation.
For eukaryotic expression of SzM protein, the *szm* gene was PCR amplified from SEZ ATCC35246 genomic DNA with primers szm_N1_R/szm_N1_F, and subcloned into the pEGFP-N vector to construct the recombinant plasmid pEGFP-N-szm. The hBMEC cells were cultured in 6-well Glass Bottom Plates (Cellvis, P06-1.5H-N) for 3-4 days until 90% confluence. Cells were replenished with Opti-MEM medium (Gibco, 31985070), and the recombinant plasmid pEGFP-N-szm and control vector pEGFP-N were transfected into hBMECs, respectively. The live-cell observation was started at 18 h after the transfection of plasmids and was continuous for 6 h. The plates were cultured in a controlled environmental chamber at 37℃ in 5% CO_2. Time-lapse images were acquired at an interval of 15 min for 360 min on an Axiom Observer Z1/7 microscope, using the Applied

Precision motorized stage (Zeiss). ZEN 2.0 and ImageJ Fiji 2.3.0 were used for image processing.

Immunofluorescence microscopy.

For the immunofluorescent micrograph in Fig. 1A, mice were sacrificed at 18 hours post SEZ infection. The brains were dissected, and paraffin embedded, and then sectioned. Briefly, the paraffin sections of brain tissue were first deparaffinized, rehydrated, and then the sections were blocked with Endogenous Peroxidase Blocking Buffer (Beyotime, p0100p) and 1% bovine serum albumin (BSA) in PBS for 1 h. Primary antibodies were used as follows: anti-CD34 rabbit mAb (Abcam, ab81289) diluted at 1:500 in PBS with 1% BSA and 0.5% Triton X-100 (PBST), and anti-SzM mAb was diluted at 1:500 in PBST. After washing with PBST 3 times, the secondary antibodies goat anti-rabbit IgG H&L (Alexa Fluor® 647) (Abcam, ab150079) and goat anti-mouse IgG H&L (Alexa Fluor® 488) (Abcam, ab150113) were diluted at 1:1,000 in PBST and incubated for 1 h at room temperature with the sections. The sections were washed and covered with ProLong® Diamond Antifade Mountant with DAPI (Invitrogen, P36966). The sections were imaged using fluorescence microscopy (Zeiss, Axio observer).

For labeling of SEZ MVs with 3,3'-dioctadecyloxacarbocyanine perchlorate (DiO) (Invitrogen, D4292), MVs from SEZ WT or from Δszm were labeled with DiO (20 μg/ml) for 30 min at 37 °C. The 30 kDa Amion ultrafiltration (Millipore, UFC9100) was used to remove the unbound dye. The labeled-MVs were filtered with 0.45 μm filter, and then used to treat WT or *pten* KO hBMECs; 10 nM CPZ or 100 nM SF1670 were added 12 h prior to MVs treatment if necessary. In *SI Appendix*, Fig. S6A, the anti-integrin β1 antibody (Abcam, ab179471) was added at 10, 1, 0.1 μg/ml 0.5 h prior to DiO-labled MVs (100 μg/ml) treatment; the commercial human IgG (Sigma, I4506) was used as negative control.

To observe the colocalization of MVs, MV-brone SzM protein and endosomes, DiO-labled MVs (500 μg/ml) were used to treat hBMECs for 9 h. After blocking with 1% BSA in PBS, anti-SzM mouse mAb (1:500) and the secondary antibody DyLight 549 goat anti-mouse IgG (H+L) (Abbkine, A23310) was used to stain the SzM protein. The endosomes were stained with anti-RaB7 rabbit mAb (1:200) (Abcam, ab126712) and the secondary antibody goat anti-rabbit IgG H&L (Alexa Fluor® 488) (Abcam, ab150077). Samples were washed and stained for 5 min with DAPI at 2 μg/ml before imaging with Fluorescent microscopy (Zeiss, Axio observer).

Intracellular protein extraction assay.

The hBMECs were seeded in 6-well cell culture plates and after treatment with SzM protein or WT MVs, the culture supernatant was discarded and cells were washed 3 times with PBS; 0.25% trypsin was then used to digest the extracellular protein for 10 min at 37 °C. Cells were lysed by RIPA and boiled for 5 min in 5 × loading sample buffer and next used for western blot analysis.

Co-Immunoprecipitation.

The hBMECs cultured in cell culture dish (100 mm) were treated with 50 μg/ml endotoxin-free SzM protein for 12 h. the culture supernatant was discarded and cells were washed 3 times with PBS; 0.25% trypsin was then used to digest the extracellular protein for 10 min at 37 °C. Cells were lysed with lysing reagent (Beyotime, P0013) and incubated with anti-SzM mAb (1:50) or anti-PTEN mAb (Abcam, ab267787) (1:30) overnight, then the mixture was incubated with protein G agarose for 2 h, all the procedures were performed at 4 °C. The beads were collected through centrifugation, washed with chilled PBS 3 times, boiled in protein loading buffer for 10 minutes, and then loaded onto an SDS-PAGE gel for immunoblot analysis with indicated antibodies.

Western blot.

Cell samples with loading buffer were subjected to SDS-PAGE, followed by transfer to a PVDF membrane (Millipore, ISEQ00010) using a semi-dry transfer apparatus (Bio-rad, 221BR). Membranes were blocked in 5% non-fat milk powder in TBS containing 0.01% Tween 20 (TBST). Primary antibodies were used and diluted as follows: 1:1000 anti-SzM mAb, 1:5000 anti-β-actin mAb (Engbody, No. AT0001), 1:2000 anti-PTEN mAb (Abcam, ab267787), 1:2000 anti-pFAK mAb (Abcam, ab81298), 1:10000 anti-p53 mAb (Abcam, ab32389), 1:50000 anti-NF2/Merlin mAb (Abcam, ab109244), 1:5000 anti-APG5L/ATG5 mAb (Abcam, ab108327), 1:2000 anti-Integrin β1 mAb (Abcam, ab179471), 1;25000 anti-SQSTM1/p62 mAb (Abcam, ab109012), 1:2000 anti-LC3b mAb (Abcam, ab192890), 1:2000 anti-Ubiquitin mAb (Abmart, M026378). After incubation with primary antibody diluted in TBST containing 1% BSA overnight at 4 °C, membranes were washed for 30 min in TBST. Secondary antibodies were diluted as follows 1:5000 HRP conjugated goat anti-rabbit IgG or goat anti-mouse IgG antibody (Invitrogen, 31460, 31430). Membranes were washed 3 times for 10 min each in TBST before adding ECL reagent (Thermo, A38555) and chemiluminescence was detected on a ChemiDoc™ Touch Imaging system (Bio-Rad).

Quantification of SzM protein in MVs.

SzM protein was diluted to final concentration of 30, 15, 7.5, 3, 1 μg/ml. The 500 μg/ml MVs and the serially diluted SzM protein were loaded to SDS-PAGE. The anti-SzM mAb (1:1000) was used as the primary antibody, and goat anti-mouse IgG (1:5000) as secondary antibody for the immunoblot. The PVDF membrane was detected on a ChemiDocTM Touch Imaging system (Bio-Rad) after incubation with ECL reagent. ImageJ Fiji was used to measure grey intensity of bands. Graphpad Prism was used to calculate the standard curve of SzM concentration and grey intensity (*SI Appendix*, Fig. S4A). There was ~5.4 μg/ml of SzM protein in 500 μg/ml MVs.

Supplementary Figures

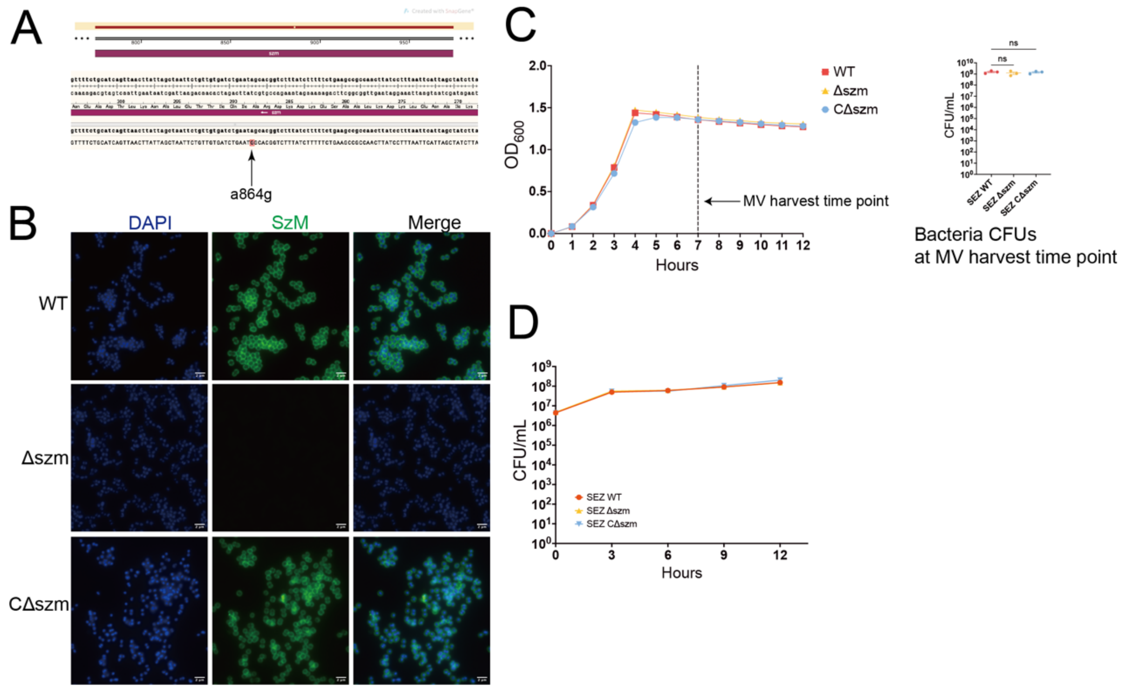

Fig S1. Characterization of SEZ wild-type strain, *szm* gene deletion strain and its complemented strain. (A) A *szm* gene with a silent point mutation at nucleotide 864 (A to G) was inserted into the native *szm* locus to complement the Δszm deletion mutant strain. **(B)** Immunofluorescence microscopy detection of SzM in wild-type SEZ (WT), *szm* gene deletion strain (Δszm), and complemented strain (CΔszm) with FITC conjugated anti-SzM mAb (scale bar = 2 μm). **(C, D)** Growth curves of indicated SEZ strains. Bacteria were cultured with Todd Hewitt broth (C) or porcine CSF (D) at 37 °C and absorbance OD_{600} or CFU was measured. The CFU of culture in THB was evaluated at MV harvest time point (7 h) to check bacterial viability (ns indicates no significant difference with one-way ANOVA).

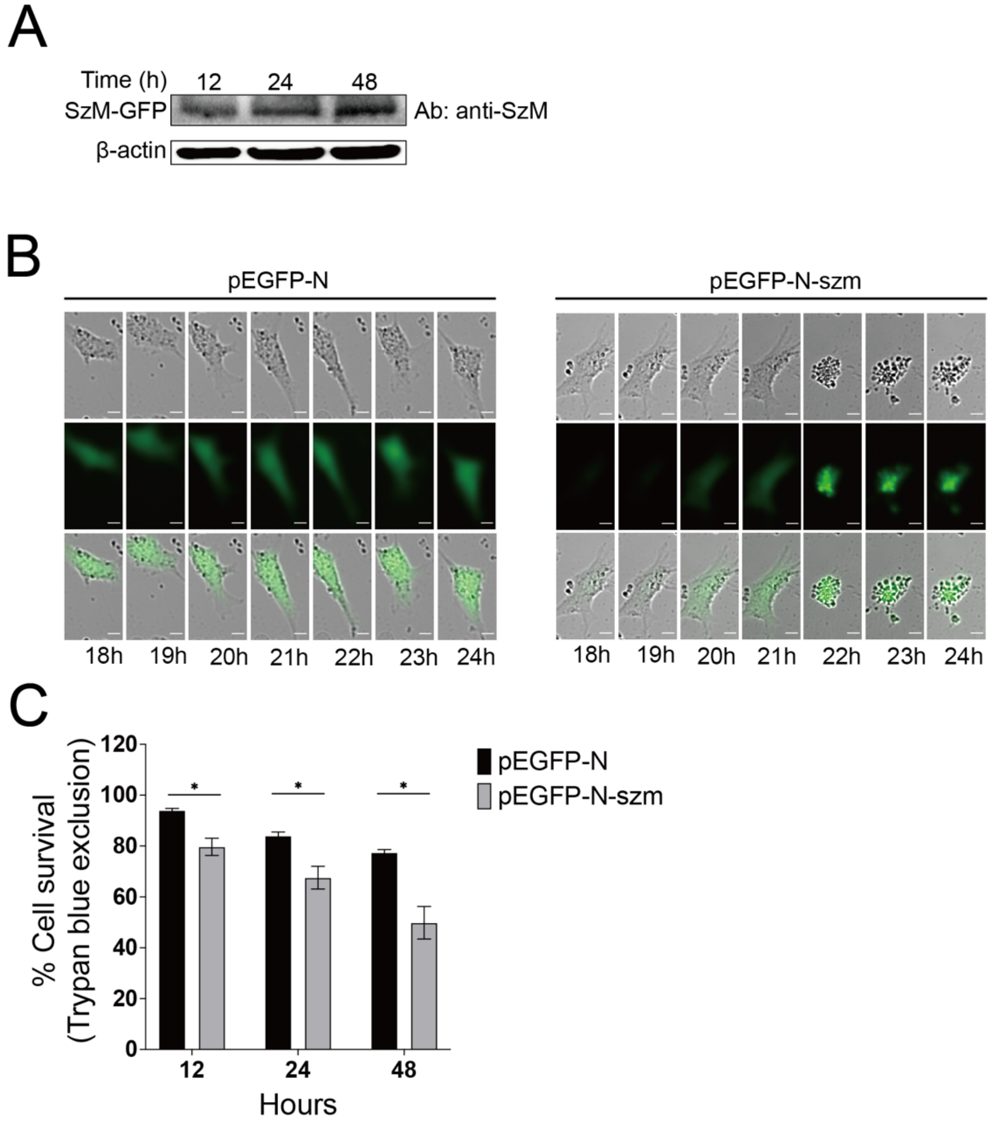

Fig S2. Intracellular SzM protein in host cells is cytotoxic. (A) Immunoblot detection of SzM fused with GFP recombinant protein (SzM-GFP protein) with anti-SzM mAb in the cell lysates of hBMECs after pEGFP-N1-SzM plasmid transfection at 12, 24, and 48 h. β-actin was used as a reference protein. **(B)** Live-cell observation of the expression of the SzM-GFP protein in the hBMECs from 18 to 24 h after pEGFP-N1-SzM plasmid transfection. GFP expressed in hBMECs after pEGFP-N1 plasmid transfection was used as the negative control. Images were acquired in brightfield (DIC) and FITC channels (scale bar = 20 μm). **(C)** The percentage of cell survival (measured by trypan blue) after pEGFP-N1-SzM or pEGFP-N plasmid transfection to hBMECs for 12, 24, and 48 h (* indicates $p < 0.05$ with unpaired Student's *t*-test).

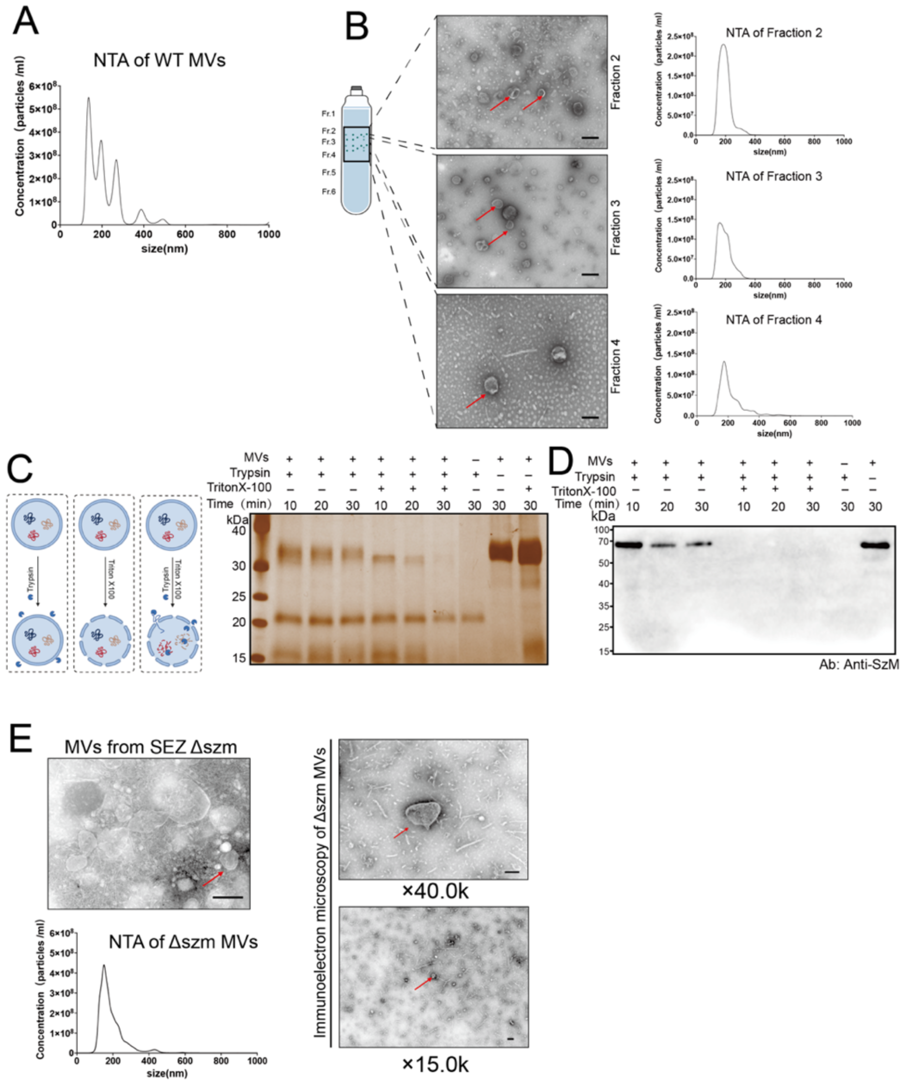

Fig S3. The MVs of SEZ contain SzM protein. (A) Nanoparticle tracking analysis (NTA) of the concentration and size of particles in the ultra-centrifugated pellet from ultra-filtered SEZ culture supernatant. **(B)** Purified MVs from fractions 2, 3, 4 after density gradient ultra-centrifugation were observed with TEM (scale bar = 200 nm) and analyzed with NTA. **(C)** Schematic of the experimental design of MVs protease sensitivity experiments. Created with BioRender.com. Purified MVs (fraction 2) were treated with trypsin +/- TritonX-100. Samples were detected by silver staining and **(D)** western blot with anti-SzM mAb**. (E)** The crude MVs isolated from the Δszm mutant were observed with TEM (scale bar = 200 nm) and analyzed with NTA. Immunonoelectron microscopy of MVs purified from Δszm and visualized with anti-SzM mAb and anti-mouse IgG colloid gold conjugated secondary antibody (scale bar = 200 nm).

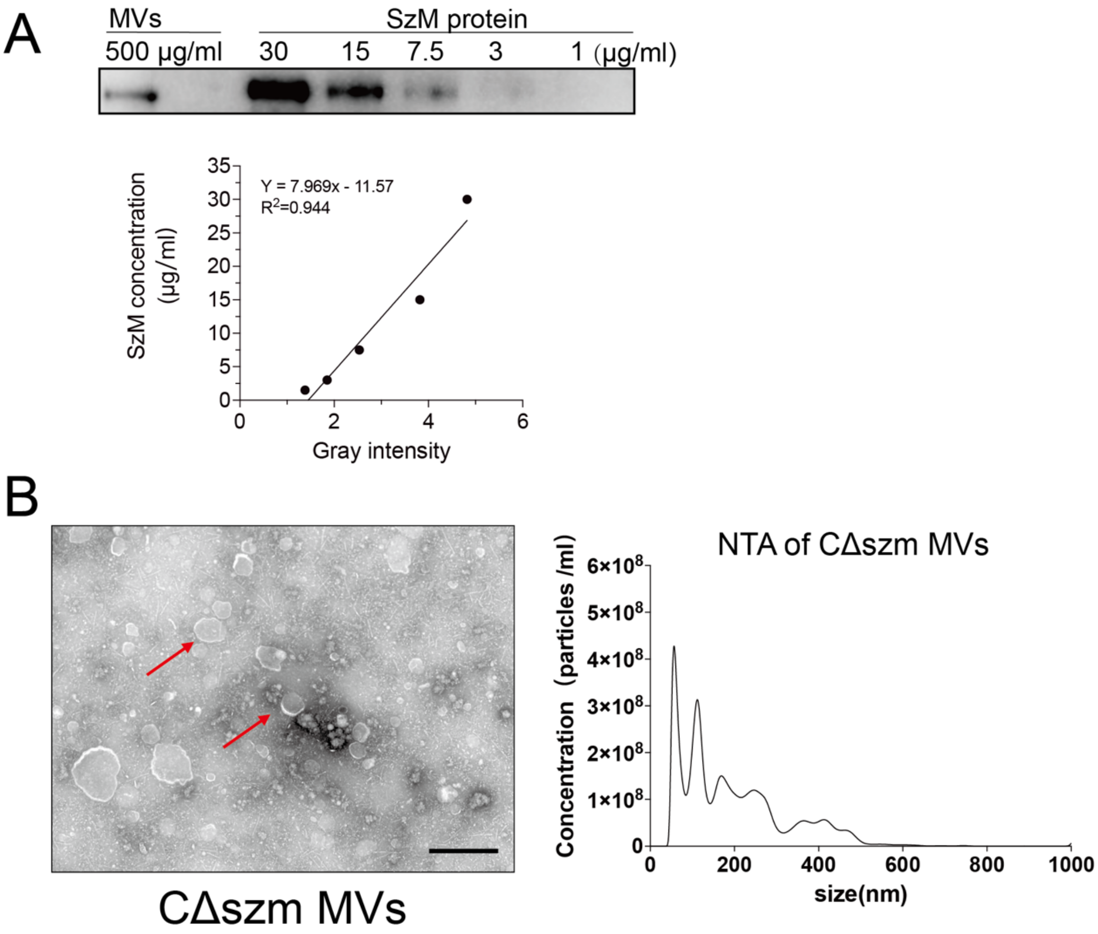

Fig S4. Quantification of SzM protein in MVs and detection of MVs isolated from CΔszm. (A) Semi-quantification of SzM in MVs by immunoblot. Gray intensity values (measured by ImageJ Fiji) of known concentrations of SzM were used to generate the standard curve. **(B)** The crude MVs from CΔszm were observed with TEM (scale bar = 200 nm). The particle concentration and size of MVs samples were measured by NTA.

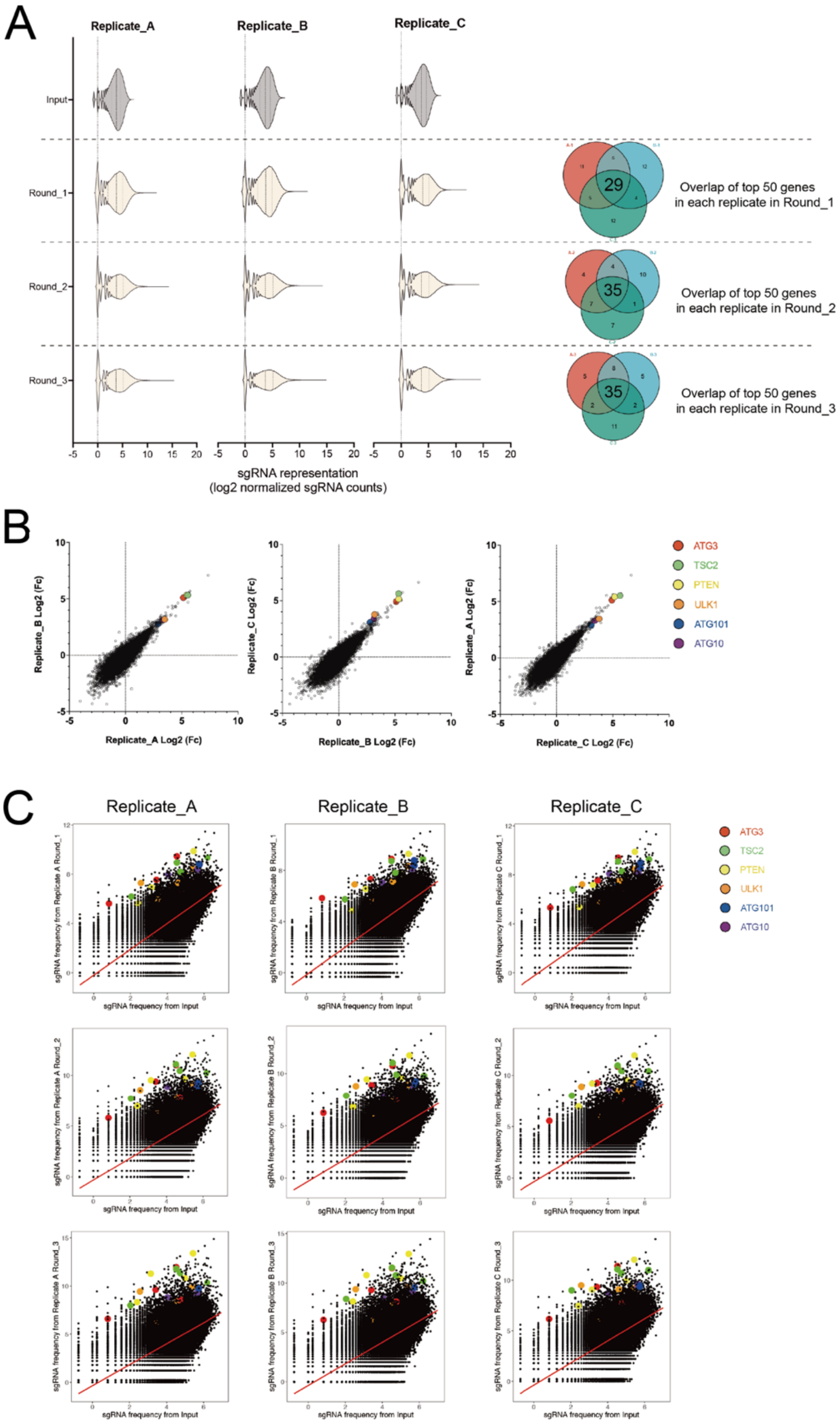

Fig S5. Data analysis of CRISPR-screen outcomes. (A) Violin plot of sgRNA count number in the input library and 3 rounds of screen outputs. The Venn diagram shows the overlap genes of the top 50 genes in 3 replicates from each round of the screen. **(B)** The

log2 fold change (log2 Fc) of the gene candidates from the 3 rounds screen in all biological replicates. The colored circles indicate the 6 enriched genes in the autophagy pathway. **(C)** Scatterplots showing enrichment of sgRNAs for SzM protein cytotoxicity screen in 3 rounds of screening with 3 parallel biological replicates. The sgRNAs targeting the same gene are highlighted with the same color and are components of the autophagy pathway. The values correspond to log2 of normalized reads counts in Dataset S4.

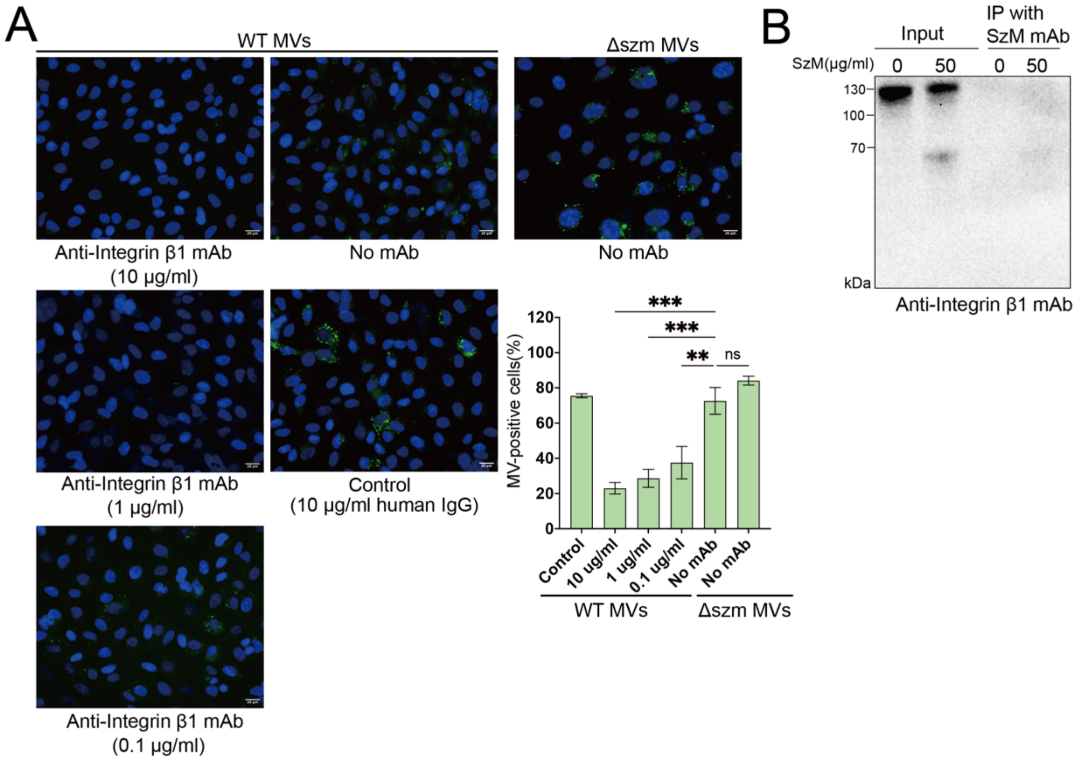

Fig S6. Integrin β1 was relevant to MVs endocytosis of hBMECs but had no interaction with the SzM protein. (A) Detection of intracellular DiO-labeled (green) MVs in hBMECs with fluorescent microscope at 9 h after exposure to 100 μg/ml MVs. The anti-integrin β1 antibody was added at indicated concentration at 0.5 h prior to MVs treatment. The commercial human IgG was used as negative control. The MV-positive cells were counted from 3 independently acquired images of each sample (***indicates $p < 0.001$, ** indicates $p < 0.01$, ns indicated no significant difference with one-way ANOVA). **(B)** The interaction detection of SzM and integrin β1 by Co-IP with anti-SzM antibody and immunoblot against anti-integrin β1 antibody. Input was the cell lysis from the hBMECs after exposure to SzM protein for 12 h.

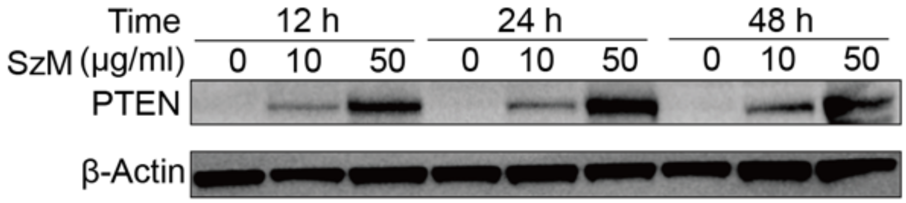

Fig S7. Immunoblot detection of PTEN in the differentiated THP-1 cells after indicated concentrations of SzM incubation at different time points (β-actin was used as reference).

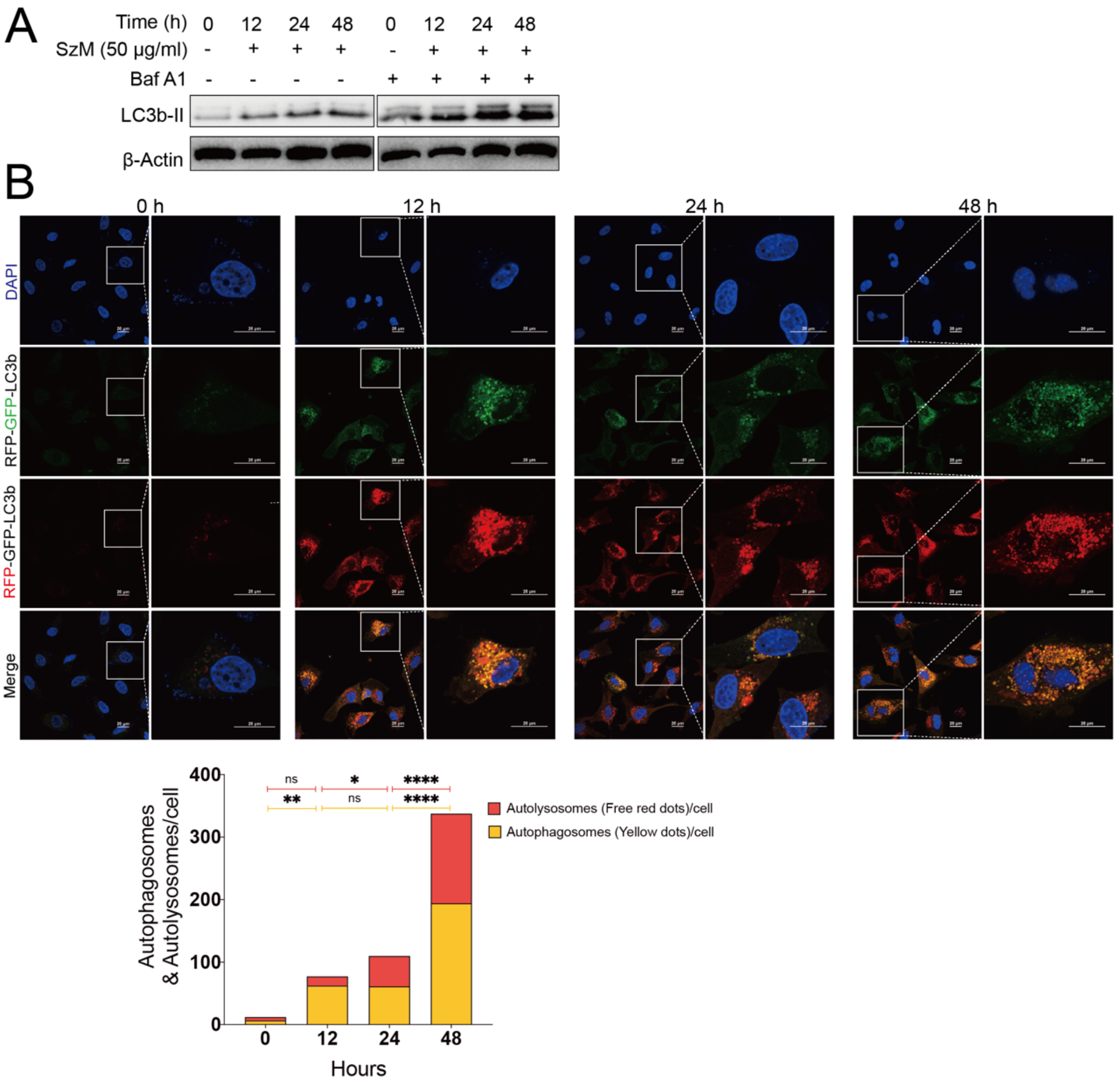

Fig S8. The autophagy flux in hBMECs after SzM protein treatment. (A) Immunoblot detection of LC3b-II after SzM incubation with or without Bafilomycin A1 (Baf A1) for indicated times. **(B)** Autophagy flux in hBMECs after SzM protein (50 μg/ml) treatment at indicated time points were detected with an RFP-GFP-LC3b fusion protein with confocal fluorescent microscopy (scale bar = 20 μm). The red (autolysosome) and yellow (autophagosome) dots were counted to evaluate formation of autolysosome and autophagosome (****indicates $p < 0.0001$, ** indicates $p < 0.01$, * indicates $p < 0.05$, ns indicates no significant difference with two-way ANOVA, red line indicates the statistical analysis of red dots, yellow line indicates the statistical analysis of yellow dots).

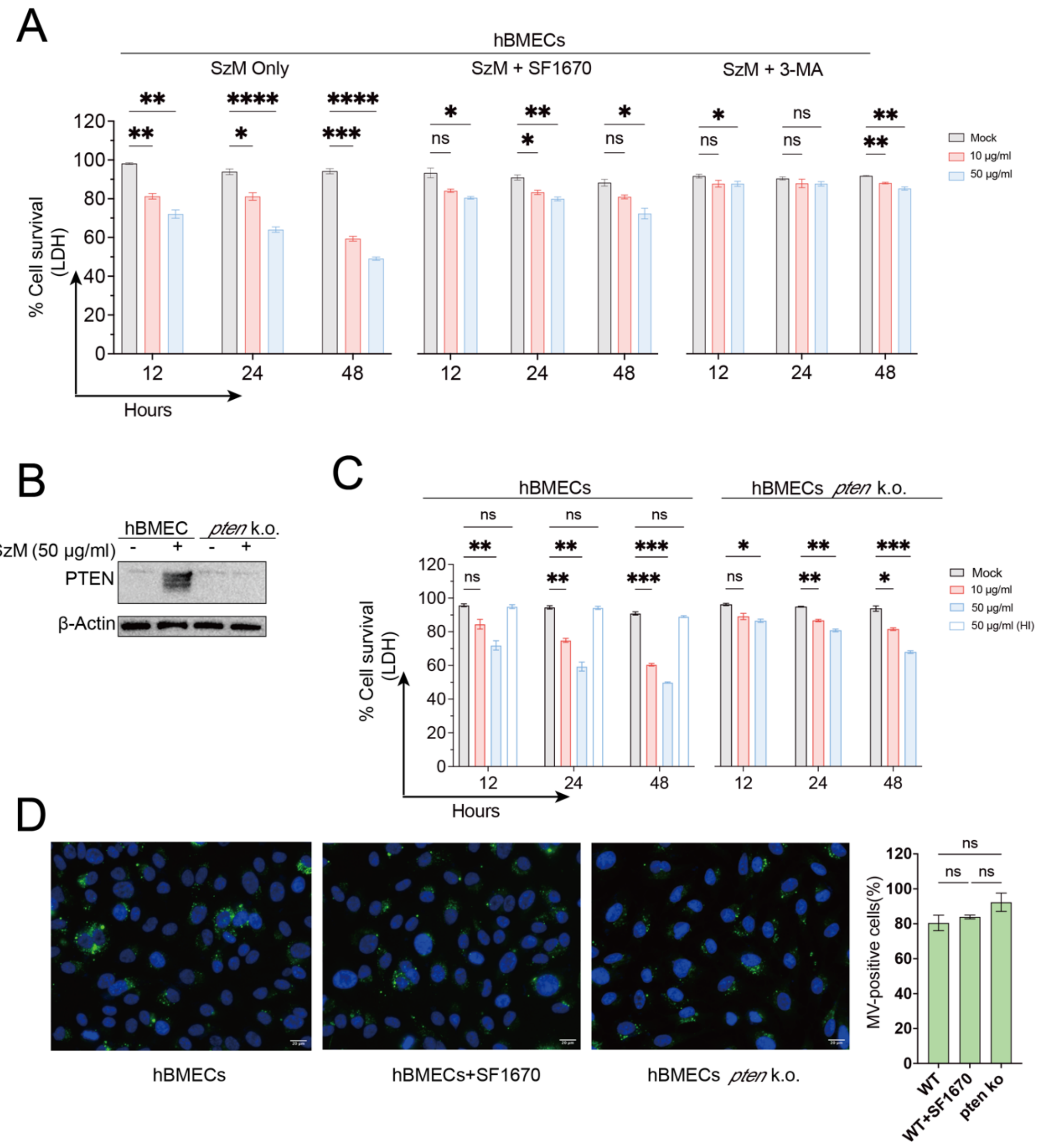

Fig S9. Pharmacological inhibition of SzM induced cytotoxicity. (A) Survival of hBMECs (LDH release) after indicated treatment (SzM protein, PTEN inhibitors SF1670 or autophagy inhibitor 3-MA) at different time points. **(B)** Validation of *pten* gene knockout in hBMECs. Immunoblot against PTEN was used to detect the PTEN expression level in wildtype and *pten* KO hBMECs after SzM protein treatment (β-actin was used as reference). **(C)** Survival of hBMECs or *pten* K.O. hBMECs (LDH release) after indicated concentrations of SzM protein (or heat inactivated [HI] SzM protein) treatment at different time points. In (A) and (C), PBS treated groups were used as mock. (n=4, **** indicates p < 0.0001, *** indicates p < 0.001, ** indicates p < 0.01, * indicates p < 0.05 and ns indicates no significant difference with two-way ANOVA). **(D)** Detection of intracellular DiO-labeled

(green) MVs in wildtype and *pten* KO hBMECs with fluorescent microscope at 9 h after exposure to 100 μg/ml MVs. The SF1670 was added 12 h prior to DiO-labeled MVs to evaluate its influence on the endocytosis of wildtype hBMECs. (The MV-positive cells were measured in 3 duplicates graphs, ns indicates no significant difference with one-way ANOVA).

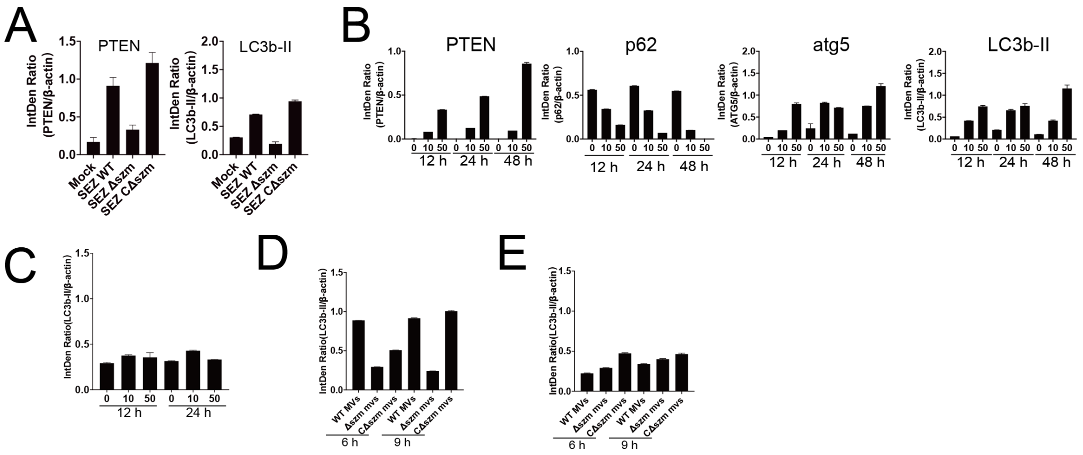

Fig S10 Gray intensity graphs generated from Fig. 6 immunoblots. (A) from (6A), **(B)** from (6B), **(C)** from (6C), **(D)** from (6F), and **(E)** from (6G). All above gray intensities were measured with ImageJ Fiji for 3 replicates and IntDen Ratio was calculated as grey intensity of LC3b-II divided by grey intensity of β-actin.

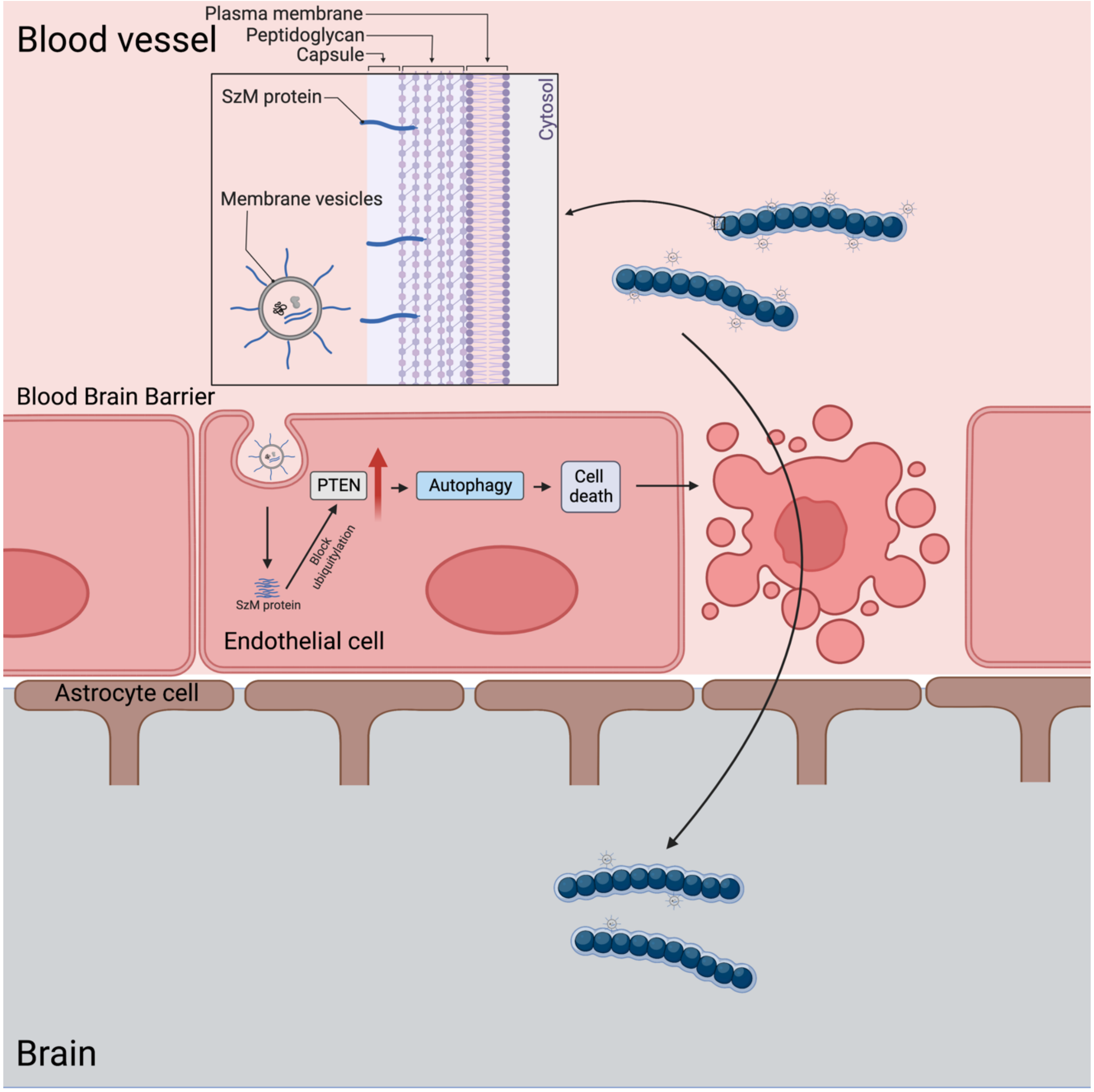

Fig S11. Schematic model of SEZ disruption of the BBB via MV delivery of SzM to CNS endothelial cells. SEZ sheds MVs containing SzM and these MVs are endocytosed by BMECs. Intracellular SzM triggers PTEN activation related autophagic cell death through blocking PTEN ubiquitylation, leading to disruption of the BBB and facilitating SEZ's paracellular traversal of the BBB into the CNS. Created with BioRender.com.

Supplementary Movies

Movie S1 (separate file). Observation of hBMECs expressing SzM-GFP fused protein with live-cell imaging (scale bar = 10μm).

Movie S2 (separate file). Observation of hBMECs expressing GFP protein with live-cell imaging (scale bar = 10μm).

Supplementary Datasets

Dataset S1 (separate file) The protein list of LC/MS outcomes of the purified MVs samples from 3 biological replicates.

Dataset S2 (separate file) The gene list from the MAGeCK analysis of the 3 biological replicates of 3 rounds of SzM protein screened hBMECs CRISPR library.

Dataset S3 (separate file) The sgRNAs list from the MAGeCK analysis of the 3 biological replicates of 3 rounds of SzM protein screened hBMECs CRISPR library.

Dataset S4 (separate file) The normalized counts of the sgRNA from the MAGeCK analysis of the 3 biological replicates of 3 rounds of SzM protein screened hBMECs CRISPR library.

Supplementary Tables

Table S1 Primers used in this research.

Primer name	Sequence (5'-3')
szm_truncated_F	aaacgacggccagtggcatgggacgccaga
szm_truncated_R	ggaattgacatgctatttcctctttccccaat
szm_N1_R	aggaaatagcatgtcaattcctctctaccc
szm_N1_F	tgaccatgattacgccatttgagcacatctggcatt
szm_down_F	gtgcggccgcaagctttgaccagctttagcagttgtagtgcc
szm_down_R	tcgcggatccgaattaggagcggctgtaaaggc
szm_up_F	tctcgagctcaagcttaatgggagcggctgtaaaggcg
szm_up_R	ggcgaccggtggatcctgaccagctttagcagttgtagtgc
Cszm_up _F	gacggccagtgaattcagagattttgtagaattcgatctcattgaggtag
Cszm_up _R	aagaccgtgccattcagatcacaacagaattagctaataagttaact
Cszm_down_F	gaatggcacggtctttatctttttctgaagccg
Cszm_down_R	cgactctagaggatccagggataaaggtatttcgcaggct
pten-ko1-F	caccgagagcgtgcagataatgaca
pten-ko1-R	aaactctcgcacgtctattactgtc

SI References

1. Z. Ma *et al.*, A streptococcal Fic domain-containing protein disrupts blood-brain barrier integrity by activating moesin in endothelial cells. *PLoS Pathog* **15**, e1007737 (2019).
2. D. Takamatsu, M. Osaki, T. Sekizaki, Thermosensitive suicide vectors for gene replacement in *Streptococcus suis*. *Plasmid* **46**, 140-148 (2001).
3. M. Radu, J. Chernoff, An in vivo assay to test blood vessel permeability. *J Vis Exp* 10.3791/50062, e50062 (2013).
4. J. G. Doench *et al.*, Optimized sgRNA design to maximize activity and minimize off-target effects of CRISPR-Cas9. *Nat. Biotechnol.* **34**, 184 (2016).
5. B. Wang *et al.*, Integrative analysis of pooled CRISPR genetic screens using MAGeCKFlute. *Nat. Protoc.* **14**, 756-780 (2019).
6. G. Yu, L. Wang, Y. Han, Q. He, clusterProfiler: an R package for comparing biological themes among gene clusters. *Omics : a Journal of Integrative Biology* **16**, 284-287 (2012).
7. H. Song *et al.*, Protection Efficacy of Monoclonal Antibodies Targeting Different Regions of Specific SzM Protein from Swine-Isolated *Streptococcus equi* ssp. *zooepidemicus* Strains. *Microbiology spectrum* 10.1128/spectrum.01742-22, e0174222 (2022).
